# A CTL − Lys immune function maintains insect metamorphosis by preventing gut bacterial dysbiosis and limiting opportunistic infections

**DOI:** 10.1186/s12915-024-01855-8

**Published:** 2024-03-06

**Authors:** Pei Xiong, Wen-Wen Wang, Xu-Sheng Liu, Yu-Feng Wang, Jia-Lin Wang

**Affiliations:** Hubei Key Laboratory of Genetic Regulation and Integrative Biology, School of Life Sciences, Central China Normal University, Wuhan, 430079 China

**Keywords:** Pupariation, Lipid metabolism, Ecdysone, C-type lectin, Lysozyme

## Abstract

**Background:**

Gut bacteria are beneficial to the host, many of which must be passed on to host offspring. During metamorphosis, the midgut of holometabolous insects undergoes histolysis and remodeling, and thus risks losing gut bacteria. Strategies employed by holometabolous insects to minimize this risk are obscure. How gut bacteria affect host insects after entering the hemocoel and causing opportunistic infections remains largely elusive.

**Results:**

We used holometabolous *Helicoverpa armigera* as a model and found low *Lactobacillus* load, high level of a C-type lectin (CTL) gene CD209 antigen-like protein 2 (*CD209*) and its downstream lysozyme 1 (Lys1) in the midgut of the wandering stage. CD209 or Lys1 depletion increased the load of midgut *Lactobacillus*, which further translocate to the hemocoel. In particular, CD209 or Lys1 depletion, injection of *Lactobacillus plantarum*, or translocation of midgut *L. plantarum* into the hemocoel suppressed 20-hydroxyecdysone (20E) signaling and delayed pupariation. Injection of *L. plantarum* decreased triacylglycerol and cholesterol storage, which may result in insufficient energy and 20E available for pupariation. Further, Lysine-type peptidoglycan, the major component of gram-positive bacterial cell wall, contributed to delayed pupariation and decreased levels of triacylglycerols, cholesterols, and 20E, in both *H. armigera* and *Drosophila melanogaster*.

**Conclusions:**

A mechanism by which (*Lactobacillus*-induced) opportunistic infections delay insect metamorphosis was found, namely by disturbing the homeostasis of lipid metabolism and reducing 20E production. Moreover, the immune function of CTL − Lys was characterized for insect metamorphosis by maintaining gut homeostasis and limiting the opportunistic infections.

**Supplementary Information:**

The online version contains supplementary material available at 10.1186/s12915-024-01855-8.

## Background

Morphological traits of larvae and adults are genetically decoupled in favor of adaptation to selective forces, which possibly explains the advantage of metamorphosis [1]. Symbiotic bacteria, such as *Lactobacillus plantarum*, positively affect many aspects of host physiology and are therefore passed on to host offspring [2, 3]. Holometabolous insects are at risk of losing symbiotic bacteria, leading to opportunistic infections, as the gut is renewed during complete metamorphosis [4, 5]. Hence, the passage of symbiotic bacteria through metamorphosis is key to ensuring vertical transmission to host offspring [5], representing a fundamental problem encountered by most holometabolous insects. To minimize the risk of opportunistic infections caused by symbiotic bacteria during metamorphosis, the strategies that holometabolous insects have evolved remain obscure.

Insect immune responses, including antimicrobial peptide (AMP) synthesis, are initiated rapidly following the recognition of microbes by pattern recognition receptors (PRRs), such as peptidoglycan recognition proteins (PGRPs) and C-type lectins (CTLs) [6, 7]. Insect immunity plays crucial roles in shaping gut microbiota and minimizing opportunistic infections [8–10]. In mosquitoes, depletion of PGRP-LD results in the upregulation of several AMPs in the midgut, thus killing a large number of gut microbes [8]. In *Drosophila*, the loss of AMPs and lysozymes (Lys) contributes to an increase in the abundance and changes in the composition of gut microbiota [9]. Downregulation of AMPs resulting from fungal infection leads to dysbiosis of gut microbiota and translocation of symbiotic bacteria from the gut to hemocoel [10]. Although PRRs, AMPs, and Lys are essential for maintaining gut homeostasis, few studies have demonstrated how immune pathways regulate gut microbiota at the effector level, especially during complete metamorphosis.

Host development, such as metamorphosis, can be modulated or influenced by gut symbiont [3, 11, 12]. The gut bacterium *Acetobacter pomorum* controls *Drosophila* developmental rate by modulating insulin or insulin-like growth factor signaling [11]. Gut bacterium *L. plantarum* promotes *Drosophila* systemic growth by influencing ecdysone and insulin signaling [3]. These indicate that symbiotic bacteria are beneficial to host development in the gut. However, Ma et al. [13] have shown that gut microbiota retards larval weight growth and serves as a burden in the beetle’s development. Gut microbiota has the potential to migrate into the hemocoel and attenuate host development, representing opportunistic pathogens [14, 15]. For example, *Enterococcus faecalis* changes its role from resident symbiont in the midgut to a pathogen in the hemocoel [14]. Injection of gut bacteria *Enterocuccus mundtii* into the hemocoel reduces larval body size and delays pupariation [15]. It has been shown that the immune activation is energetically costly, leading to a decrease in growth and nutrient stores [16]. The mechanisms by which opportunistic infections affect host development, especially insect metamorphosis, remains largely elusive, although gut microbial dysbiosis and penetration of gut microbes into the circulation sometimes occur [10, 17].

The steroid hormone ecdysone is not produced and stored prior to release, but is produced on demand from its precursor cholesterol esters stored in lipid droplets (LDs) [18]. As an endocrine regulator, ecdysone and its active form 20-hydroxyecdysone (20E) not only trigger metamorphosis [19], but positively regulate insect immunity [20]. Activation of PGRP-SC2 or AMP expression extends *Drosophila* lifespan [21, 22], suggesting that immunity affects the endocrine system and development. Nunes et al. [23] have revealed that ecdysone signaling impacts pupariation by modulating the immune system, emphasizing the importance of the association between the endocrine system and immunity.

The lepidopteran insect *Helicoverpa armigera* is one of the most destructive and polyphagous pests worldwide [24]. Here, we used *H. armigera* as a model and compared the profiles of bacteria and immunity-related genes in the midgut between the feeding and wandering stages. We found that 20E enhanced the expression of midgut *CD209 antigen-like protein 2* (*CD209*, as a CTL, GenBank no. XM_021344387) and its downstream gene *Lys1* (GenBank no. XM_049840569). RNAi-mediated depletion of CD209 or Lys1 abolished the inhibitory capacity toward midgut *Lactobacillus* during the wandering stage. Translocation of overgrowing *Lactobacillus* and its possibly associated peptidoglycan (PGN) from the midgut to hemocoel led to the decreased triacylglycerol (TAG) and cholesterol storage, thereby reducing the availability of energy and ecdysone for pupariation. This study revealed the importance of CTL − Lys in gut homeostasis, which was crucial for minimizing the risk of opportunistic infections and ensuring timely metamorphosis.

## Results

### A significant decrease in *Lactobacillus* load in the midgut transitioned from the feeding to wandering stage

**Fig. 1:**
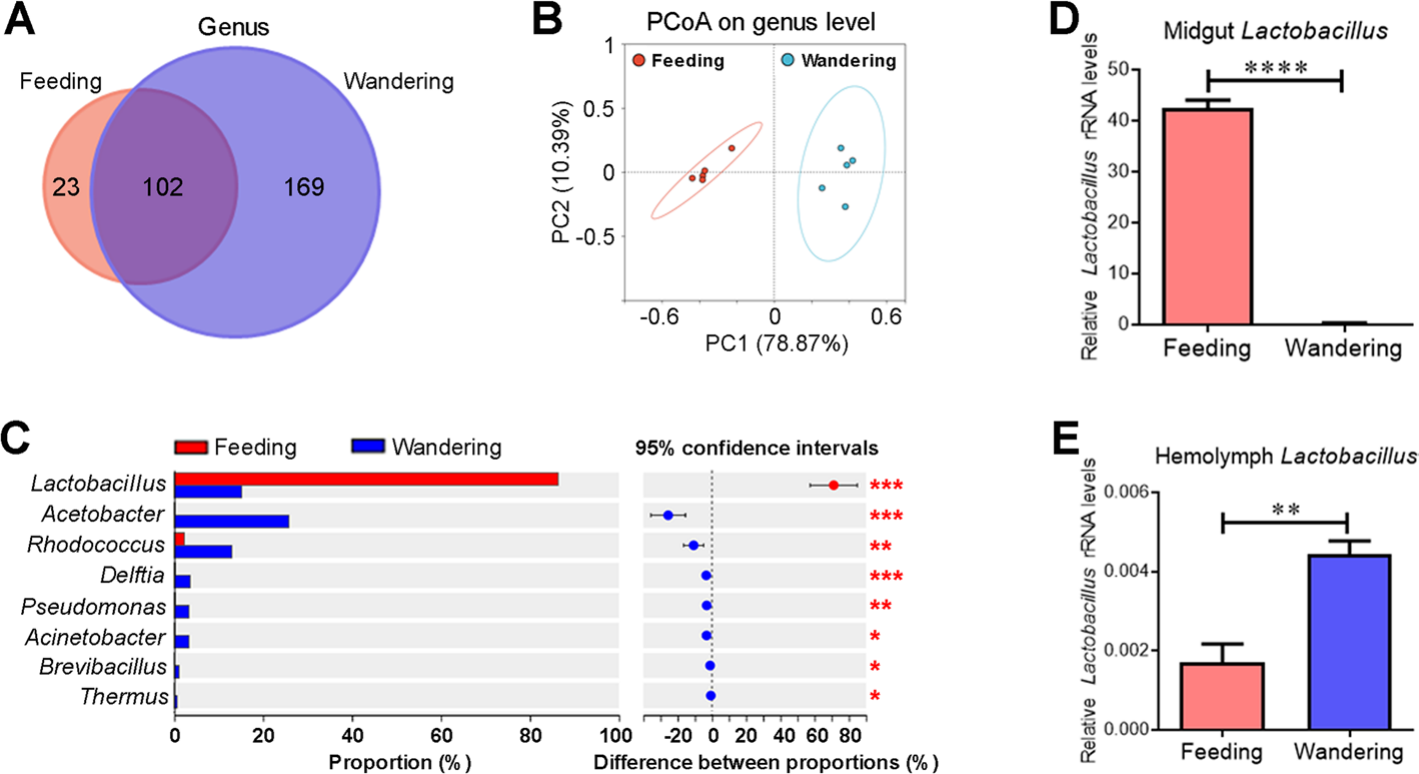
The abundance of *Lactobacillus* is significantly decreased in the midgut and significantly increased in hemolymph when larvae transition from the feeding to wandering stage. **A** Venn diagram of bacterial genus abundance in the larval midgut. Midguts were collected from sixth-instar larvae at 24 h PE (feeding) and 72 h PE (wandering). **B** Principal coordinates analysis (PCoA) of bacterial communities from the midgut based on genus level. **C** Relative abundance (%) of bacterial genera between the feeding and wandering stages. **D**, **E** Quantification of midgut *Lactobacillus* (**D**) and hemolymph *Lactobacillus* (**E**) in the feeding and wandering stages. Quantification was by qPCR analysis using primers of *Lactobacillus*-specific 16S rRNA gene. Statistical differences were analyzed using the Mann–Whitney *U* test or Student’s *t* test (*p < 0.05, **p < 0.01, ***p < 0.001, and ****p < 0.0001)

We have previously defined the developmental stages of *H. armigera* [25]. Sixth-instar at 24 h post-ecdysis (PE) is an active feeding stage, and sixth-instar at 72 h PE represents a wandering stage (the initial stage of metamorphosis). To compare the composition and diversity of microbiota, 16S rRNA gene sequencing was performed using the midguts of sixth-instar larvae at 24 h PE (feeding stage) and 72 h PE (wandering stage). In total, 455 operational taxonomic units (OTUs) were obtained, which were annotated into 24 phyla, 49 classes, 113 orders, 183 families, 294 genera, and 382 species (Additional file 1: Table S1). The number of bacterial genera in the wandering stage was higher than that in the feeding stage, with 169 unique genera in the former, 23 unique genera in the latter, and 102 genera shared (Fig. 1A).

Principal coordinate analysis (PCoA, Bray–Curtis) revealed that the structure of midgut microbiota is different between the feeding and wandering stages (Fig. 1B). Of the most abundant 8 genera, the abundance of *Lactobacillus* decreased the most, from 86.34% in the feeding stage to 15.15% in the wandering stage (Fig. 1C). Quantitative PCR (qPCR) revealed that the load of midgut *Lactobacillus* largely decreased, whereas the load of hemolymph *Lactobacillus* dramatically increased when larvae transitioned from the feeding to wandering stage (Fig. 1D, E). These results are consistent with our previous findings that the abundance of Lactobacillaceae in hemolymph is markedly elevated after larvae enter the wandering stage [15]. Similarly, the total bacterial load largely decreased in midgut and dramatically increased in hemolymph when larvae entering from the feeding to wandering stage (Additional file 2: Fig. S1).

### A significant increase of CD209 and Lys1 expression in the midgut entering from the feeding to wandering stage

**Fig. 2:**
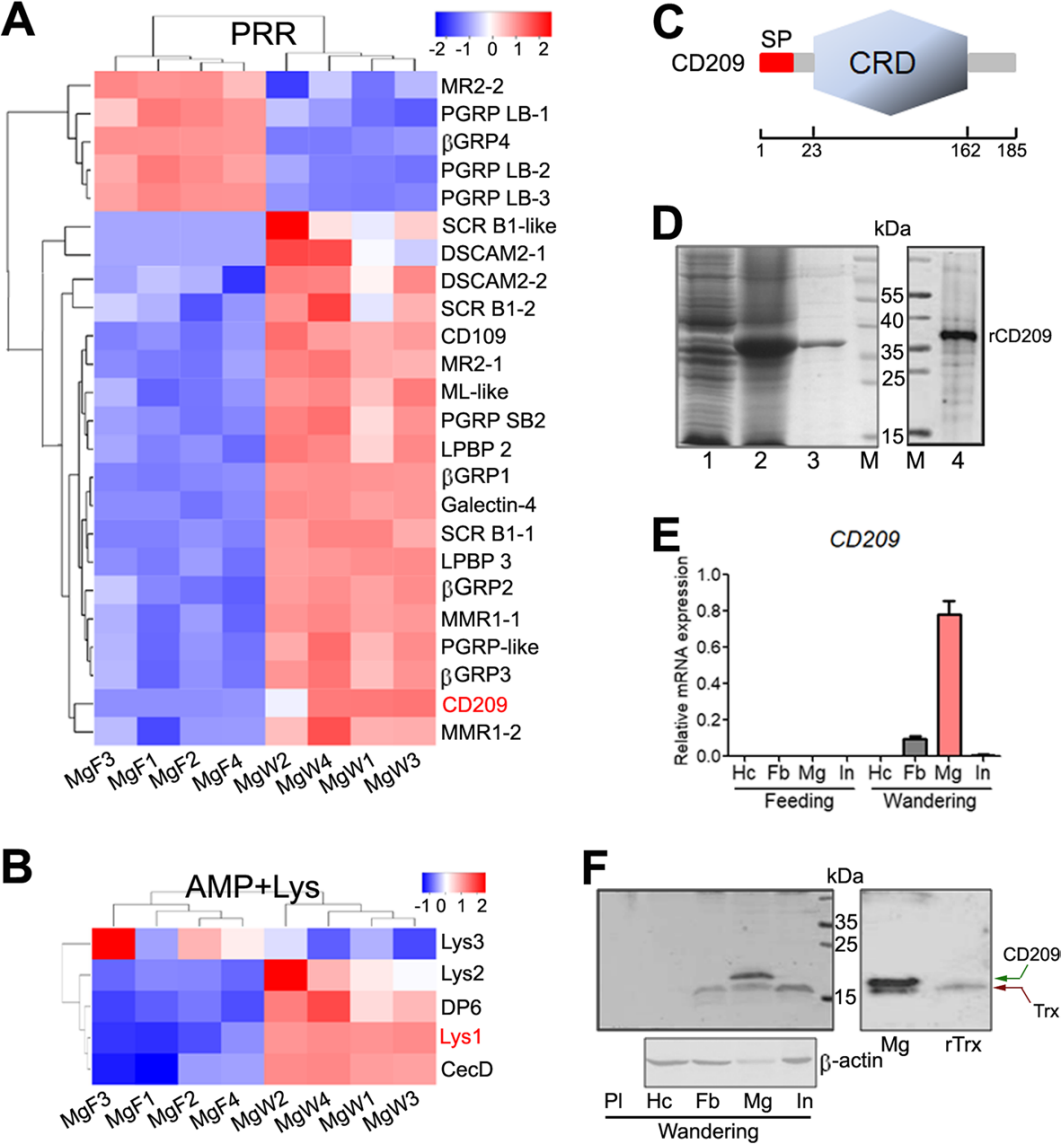
CD209 is dominantly expressed in the midgut of the wandering stage. **A**, **B** Heatmap showing the expression profiles of *PRRs* (**A**), *AMPs*, and *Lys* (**B**) in the midgut between the feeding (MgF) and wandering (MgW) stages. *CD209* and *Lys1* are highlighted in red. **C** Predicted domain architecture of CD209 protein. Characterization of a predicted signal peptide (SP, 1–19 aa) and a carbohydrate recognition domain (CRD, 23–162 aa) from http://smart.embl.de/ website. **D** Expression and purification of recombinant CD209 (rCD209), as well as rCD209 recognized by the anti-CD209 antiserum. Lanes 1 and 2, crude protein extracts from bacteria (carrying the *CD209*-pET32a plasmid) before (lane 1) and after (lane 2) IPTG induction. Lane 3, purified rCD209 protein. Lane 4, rCD209 recognized by the anti-CD209 antiserum. **E** Spatial and temporal expression profiles of *CD209* analyzed by RT-qPCR. Total RNAs were extracted from sixth-instar larvae at 24 h PE (feeding) and 72 h PE (wandering). **F** Tissue-specific expression of CD209 detected using western blotting. Antiserum against CD209 or β-actin was used for detection. Protein samples were collected from the wandering stage. Pl, plasma. Hc, hemocytes. Fb, fat bodies. Mg, midgut. In, integument

To obtain differentially expressed genes (DEGs), we performed transcriptomic sequencing of midguts from sixth-instar larvae at 24 and 72 h PE. A total of 5429 DEGs were identified, including 2712 up- and 2717 downregulated genes (Additional file 1: Table S2). Of the 5429 DEGs, 87 immunity-related DEGs were screened, including 24 genes encoding PRRs, five encoding AMPs and Lys, 12 involved in signaling, 37 encoding serine proteases, and nine encoding serpins (Additional file 1: Table S3). The transcript abundance of most of the PRR, AMP, and Lys genes increased during the wandering stage compared with the feeding stage (Fig. 2A, B).

Among PRR, AMP, and Lys genes, *CD209* and *Lys1* exhibited their respective maximal increase (Additional file 1: Table S3). CD209 contains a signal peptide and a carbohydrate recognition domain (CRD) (Fig. 2C), representing a single-CRD CTL. To analyze its function, we expressed and purified recombinant CD209 (rCD209, Fig. 2D; Additional file 3). The rCD209 has an expected molecular weight of ~ 39 kDa, including ~ 18 kDa N-terminal recombinant thioredoxin (rTrx, His-tagged). The antiserum against rCD209 could recognize the recombinant protein specifically (Fig. 2D; Additional file 3). RT-qPCR analysis indicated that *CD209* was dominantly expressed in the midgut of the wandering stage (Fig. 2E); this result was validated by western blotting (Fig. 2F; Additional file 3). Considering that the anti-CD209 antiserum could recognize rTrx, the lower band detected in the fat body, midgut, and integument might be endogenous Trx protein (Fig. 2F; Additional file 3).

### CD209 and Lys1 expression in the midgut during the wandering stage is elevated by 20E

In insect hemolymph, 20E titers are significantly higher at the wandering stage than that at feeding stage of the last instar larvae [26]. Because the transcript abundance of *CD209* and *Lys1* increased during the wandering stage (Fig. 2A, B), we hypothesized that endogenous 20E might be involved. To test the hypothesis, we injected 20E into the hemocoel of sixth-instar larvae at 48 h PE, when endogenous 20E titers are low [26]. RT-qPCR analysis revealed that at 24 h post-20E injection, *CD209* and *Lys1* expression was significantly elevated in the larval midgut (Additional file 2: Fig. S2A, B). Western blot analysis indicated that CD209 expression in the midgut increased significantly at 24 h post-20E injection (Additional file 2: Fig. S2C, D; Additional file 3). Thus, endogenous 20E contributes to increased levels of CD209 and Lys1 during the wandering stage.

### CD209 depletion results in a significant increase of *Lactobacillus* load in the midgut and hemolymph during the wandering stage

**Fig. 3:**
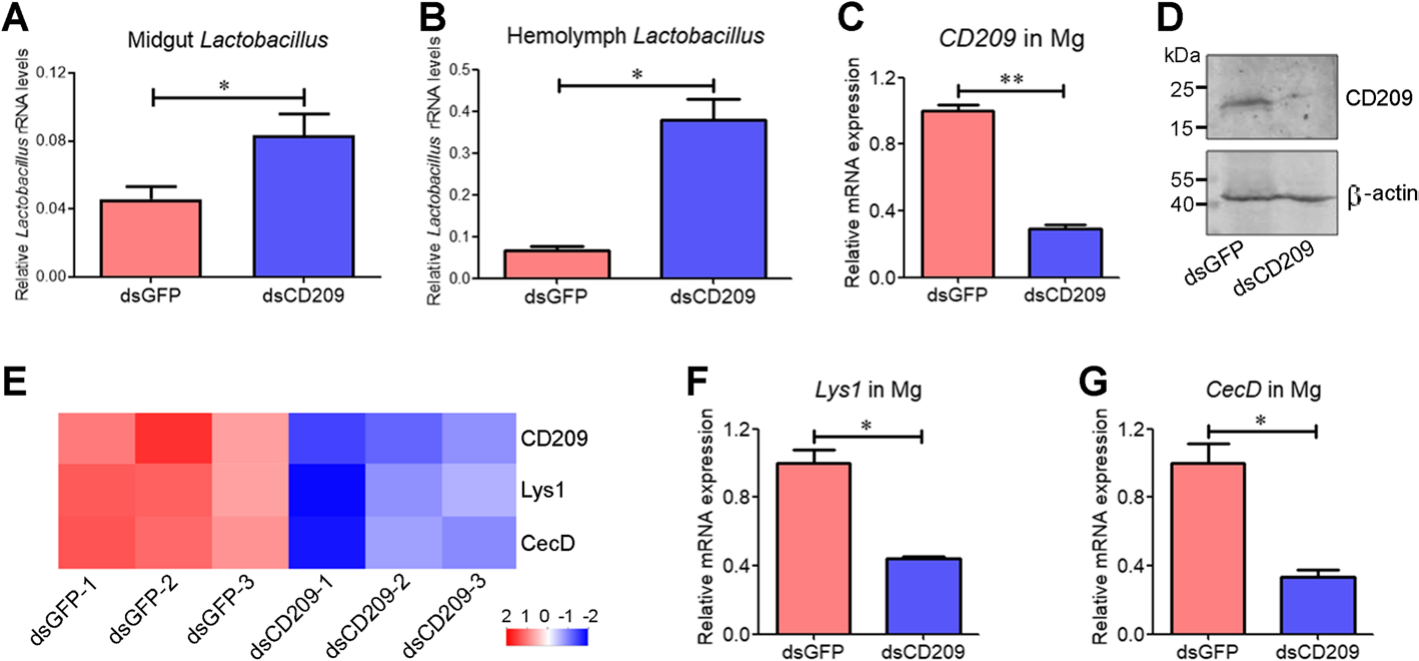
CD209 depletion increases *Lactobacillus* load and suppresses the expression of *Lys1* and *CecD*. **A**, **B** Quantification of midgut *Lactobacillus* (**A**) and hemolymph *Lactobacillus* (**B**) in larvae treated with ds*CD209* or ds*GFP*. The midgut and hemolymph were collected from sixth-instar larvae at 72 h PE. **C**, **D** Depletion of CD209 in the midgut (Mg) validated by RT-qPCR (**C**) and western blotting (**D**). The antiserum against CD209 or β-actin was used for detection. **E** Heatmap showing that DEGs encoding CD209, Lys1, and CecD were downregulated in CD209-depleted midgut. **F**, **G** RT-qPCR analysis confirming inhibition of *Lys1* (**F**) and *CecD* (**G**) expression in the CD209-depleted midgut. Statistical differences were analyzed using Student’s *t* test (*p < 0.05 and **p < 0.01)

Some studies have indicated that CTLs have antibacterial activities and play crucial roles in maintaining gut microbial homeostasis [27, 28]. Enhanced CD209 expression and decreased *Lactobacillus* load in the midgut led us to hypothesize that CD209, as a CTL, may contribute to the suppression of *Lactobacillus*. To test this hypothesis, we treated fourth-instar larvae with *CD209* dsRNA (ds*CD209*) or green fluorescent protein (*GFP*) dsRNA (ds*GFP*) and analyzed *Lactobacillus* load in sixth-instar at 72 h PE. The *Lactobacillus* load in the midgut significantly increased after CD209 depletion (Fig. 3A). Moreover, CD209 depletion increased the *Lactobacillus* load in the hemolymph (Fig. 3B). Efficient depletion of CD209 in the midgut of sixth-instar larvae at 72 h PE was confirmed by RT-qPCR and western blot analyses (Fig. 3C, D; Additional file 3).

We plated midgut homogenates of the feeding stage on deMan Rogosa Sharpe (MRS) agar plates, and picked and sequenced single colonies to characterize a gram-positive *L. plantarum* strain (GenBank no. NR_104573). *L. plantarum* exists as a single bacterium with short rod morphology. The bacteria can organize in pairs or in small chains of 3 − 4 cells (Additional file 2: Fig. S3A). Besides, rCD209 directly bound to *L. plantarum* independent of calcium ions (Additional file 2: Fig. S3B; Additional file 3). These results confirmed that CD209 senses *Lactobacillus* and functions in restricting *Lactobacillus* during the wandering stage.

### CD209 depletion largely decreases the expression of Lys1, which is essential for the suppression of *Lactobacillus* during the wandering stage

CTLs exert their antibacterial effect by regulating the expression of downstream effectors, such as AMPs [28, 29]. Since CD209, as a CTL, functions in suppression of *Lactobacillus*, we wondered whether CD209-controlled effectors were involved. To screen these effectors, we performed transcriptomic expression profiling of the midgut in response to CD209 depletion. A total of 2012 DEGs were identified in the CD209-depleted midgut, including 1301 up- and 711 downregulated DEGs (Additional file 1: Table S4). Of the 2012 DEGs, 52 immunity-related DEGs were screened, including 21 genes encoding PRRs, three encoding AMPs and Lys, six involved in signaling, 21 encoding serine proteases, and one encoding serpin (Additional file 1: Table S5). Consistent with the expression profile of *CD209*, *Lys1* and cecropin D (*CecD*) expression largely increased in the midgut entering from the feeding stage to wandering stage (Fig. 2B). Here, *Lys1* and *CecD* expression largely decreased in response to CD209 depletion (Fig. 3E). Depletion of CD209 in the midgut was confirmed by transcriptomic analysis. RT-qPCR analysis further confirmed that CD209 depletion inhibited the expression of *Lys1* and *CecD* in the midgut of sixth-instar larvae at 72 h PE (Fig. 3F, G).

**Fig. 4:**
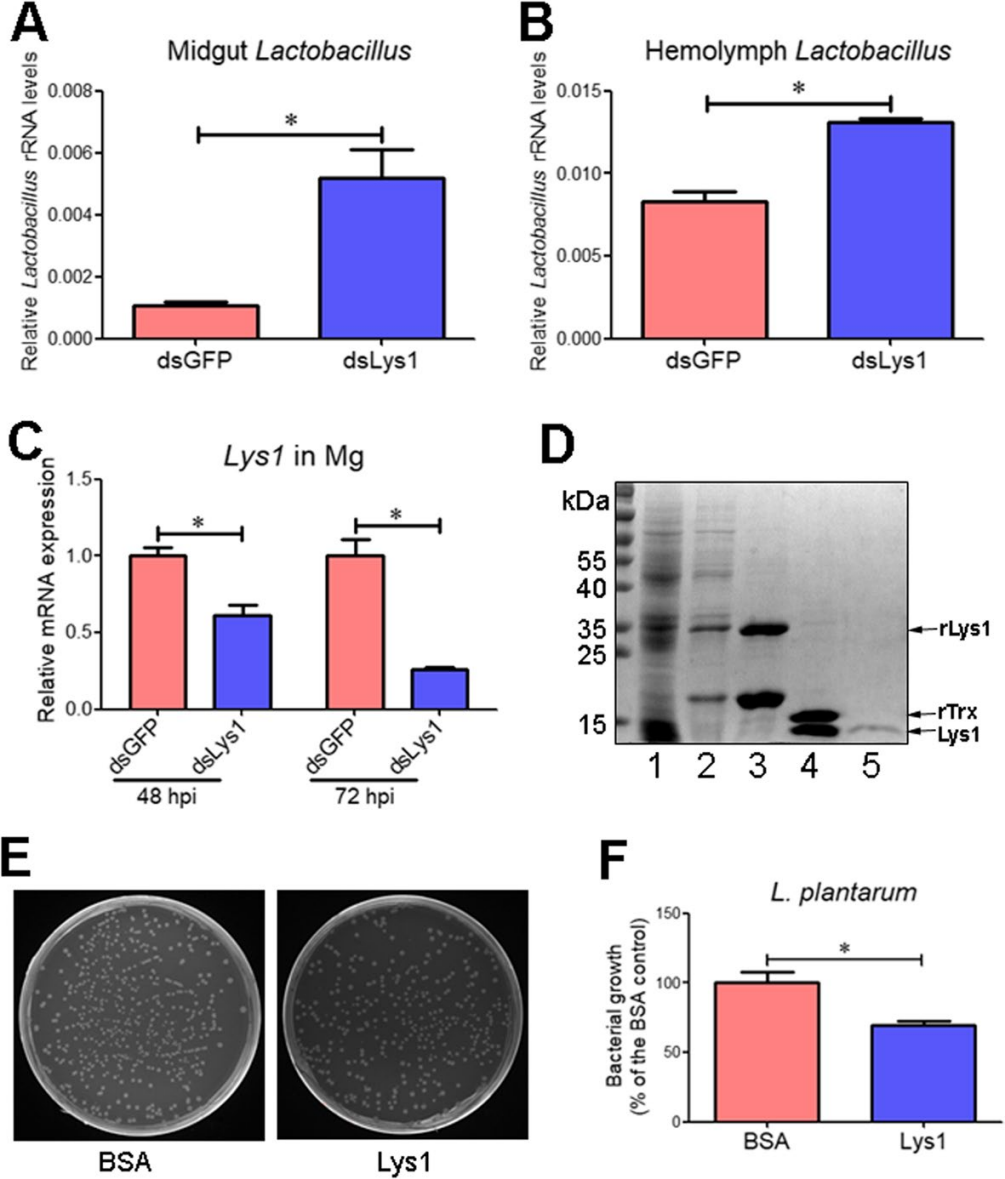
Lys1 contributes to the suppression of *Lactobacillus*. **A**, **B** Quantification of midgut *Lactobacillus* (**A**) and hemolymph *Lactobacillus* (**B**) in larvae treated with ds*Lys1* or ds*GFP*. Midguts and hemolymph were collected from sixth-instar larvae at 72 h PE. **C** Depletion of *Lys1* in the midgut at 48 and 72 h post-ds*Lys1* injection (hpi) validated by RT-qPCR. **D** Expression and purification of Lys1 mature peptide (Lys1). Lanes 1 and 2, crude protein extracts from bacteria (carrying the *Lys1*-pET32a plasmid) before (lane 1) and after (lane 2) IPTG induction. Lane 3, purified rLys1 protein. Lane 4, purified rLys1 cleaved by enterokinase. The two bands represent rTrx and Lys1. Lane 5, purified Lys1. **E** Representative MRS agar plates showing *L. plantarum* colonies after each treatment. *L. plantarum* were incubated with Lys1 or BSA at 25°C with agitation for 1 h, followed by spreading the mixture on MRS agar plates. **F** The number of *L. plantarum* colonies for each treatment was determined. Bacterial growth was exhibited as the ratio of viable colonies against BSA controls. Statistical differences were analyzed using Student’s *t* test (*p < 0.05)

To test whether CD209-controlled Lys1 was involved in the suppression of *Lactobacillus*, we treated fourth-instar larvae with *Lys1* dsRNA (ds*Lys1*) or ds*GFP*. The *Lactobacillus* load in the midgut of sixth-instar larvae at 72 h PE significantly increased after Lys1 depletion (Fig. 4A). Moreover, Lys1 depletion increased the *Lactobacillus* load in the hemolymph of sixth-instar larvae at 72 h PE (Fig. 4B). RT-qPCR analysis confirmed the depletion of *Lys1* in the midgut at 48 and 72 h post-dsRNA injection (Fig. 4C).

Lys1 mature peptide (21–142 aa) shows 97% identity with the corresponding sequence (21–135 aa) of *H. armigera* C-type Lys (GenBank no. ABF51015; Additional file 2: Fig. S4). Considering that insect C-type Lys owns antibacterial activity [30, 31], we wondered whether Lys1 directly inhibited the growth of *L. plantarum*. Therefore, we expressed and purified recombinant Lys1 mature peptide (rLys1; Fig. 4D; Additional file 3). The rTrx was removed by digestion with enterokinase, and mature Lys1 (Lys1, ~ 14 kDa) was obtained. The growth of *L. plantarum* was significantly suppressed after incubation with Lys1 (Fig. 4E, F). These results revealed the contribution of CD209-controlled Lys1 in restricting *Lactobacillus* growth during the wandering stage.

### Depletion of CD209 or Lys1 delays pupariation, which is at least partially due to the translocation of overgrowing *Lactobacillus* from the midgut to hemocoel

**Fig. 5:**
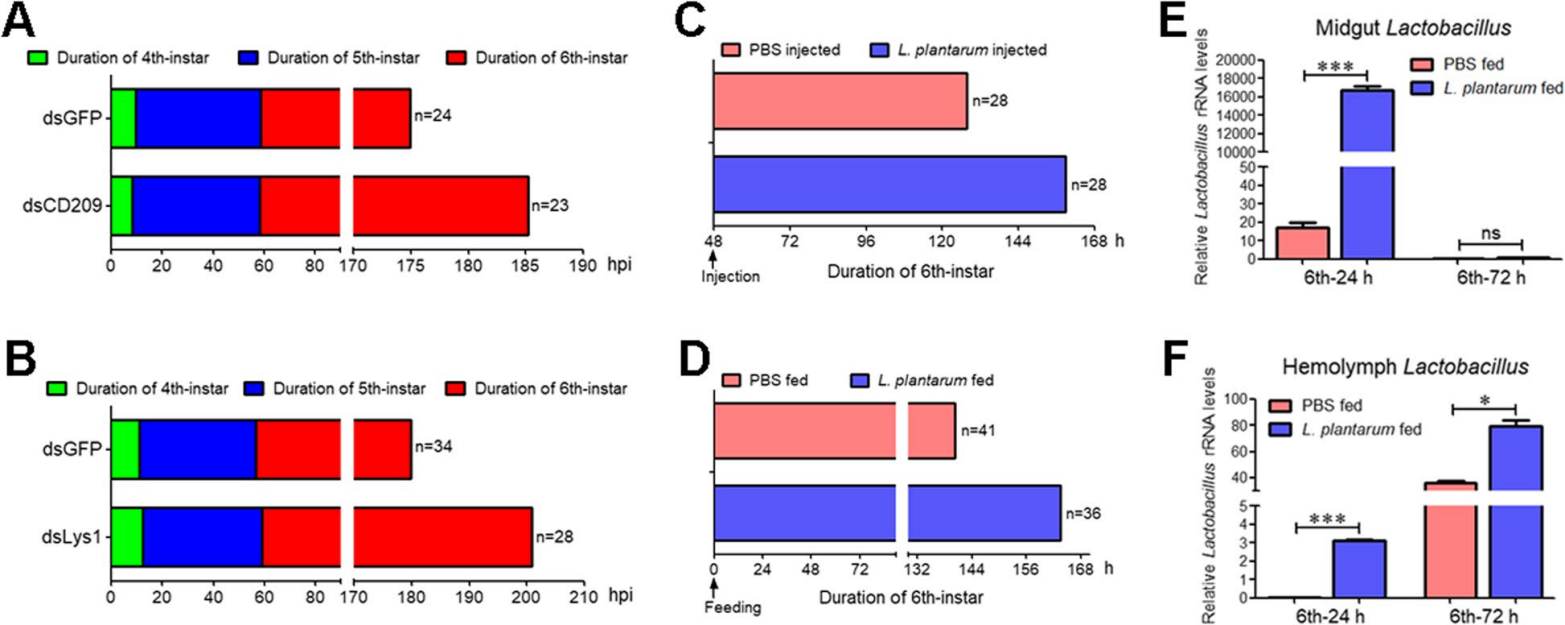
Delayed pupariation in CD209- or Lys1-depleted larvae, as well as in *L. plantarum*-injected or *L. plantarum*-fed larvae. **A**, **B** Prolonged duration of sixth-instar in CD209-depleted (**A**) or Lys1-depleted (**B**) larvae. The durations of fourth-, fifth-, and sixth-instar were measured individually and the average time was calculated. The ds*GFP* was injected as control. **C** Prolonged duration of sixth-instar in *L. plantarum*-injected larvae. *L. plantarum*-suspended PBS or PBS (control) was injected into the hemocoel of sixth-instar larvae at 48 h PE. **D** Prolonged duration of sixth-instar in *L. plantarum*-fed larvae. Axenic larvae were fed on an artificial diet supplemented with *L. plantarum*-suspended PBS or PBS (control) at the beginning of the sixth-instar stage. **E**, **F** Quantification of *Lactobacillus* in the midgut (**E**) and hemolymph (**F**) from *L. plantarum*-fed sixth-instar larvae at 24 h PE (6th-24 h) and 72 h PE (6th-72 h). Statistical differences were analyzed using Student’s *t* test (*p < 0.05 and ***p < 0.001). ns, no significant difference

During gene depletion assays, we found that depletion of either CD209 or Lys1 delayed larval pupariation. The duration of fourth- or fifth-instar between the ds*CD209*/ds*Lys1*-injected group and the control group exhibited no obvious differences; however, the CD209- and Lys1-depleted groups showed delayed pupariation for an average of ~ 11 and 19 h, respectively (Fig. 5A, B). Because depletion of either CD209 or Lys1 significantly increased the *Lactobacillus* load in the hemolymph, we wondered whether bacterial injection into the larval hemocoel delayed pupariation. Injection of *L. plantarum* into the hemocoel of sixth-instar larvae at 48 h PE delayed pupariation for an average of ~ 31 h (Fig. 5C).

Studies have shown that increased loads of bacteria in the midgut will translocate into the hemocoel [10]. Indeed, CD209 or Lys1 depletion increased the *Lactobacillus* load in both the midgut and hemolymph. We therefore hypothesized that the increase in *Lactobacillus* in hemolymph might come from the midgut where the bacteria overgrew. To test this hypothesis, we first obtained axenic larvae by treatment with oral antibiotics. The efficacy of bacterial elimination was confirmed by plating gut homogenates on Luria–Bertani (LB) agar plates (Additional file 2: Fig. S5A) and conducting PCR using the universal primers for the 16S rRNA gene (Additional file 2: Fig. S5B; Additional file 3). Subsequently, the axenic larvae at the beginning of sixth-instar were reared on artificial diet, supplemented with or without *L. plantarum*. Larvae fed with *L. plantarum* delayed pupariation for an average of ~ 23 h (Fig. 5D). The large increase in *Lactobacillus* load in the midgut at 24 h post-*L. plantarum*-fed was confirmed (Fig. 5E). *Lactobacillus* could hardly be detected in the midgut at 72 h post-*L. plantarum*-fed (Fig. 5E). This may be due to the cessation of feeding by the wandering larvae and elevated levels of CD209 and Lys1. Correspondingly, the large increase in *Lactobacillus* load in the hemolymph at 24 and 72 h post-*L. plantarum*-fed was confirmed (Fig. 5F). Thus, an increased load of *Lactobacillus* in the midgut translocates into the hemocoel and mimics the phenotype caused by CD209 or Lys1 depletion.

### Depletion of CD209 or Lys1 or translocation of *Lactobacillus* from the midgut to hemocoel inhibits 20E signaling

Because ecdysone promotes larval metamorphosis [19], we measured 20E titers in the hemolymph of wandering stage. In the hemolymph, 20E titers exhibited an overall downward trend upon CD209 or Lys1 depletion (Additional file 2: Fig. S6A). *L. plantarum* injection significantly suppressed 20E titers in the hemolymph from sixth-instar larvae at 96 h PE, whereas *L. plantarum* feeding significantly inhibited 20E levels from sixth-instar larvae at 72 h PE (Additional file 2: Fig. S6B, C).

Because *broad* and hormone receptor 3 (*HR3*) are 20E primary response genes and broad dictates pupal commitment [32, 33], we further tested their expression. CD209 or Lys1 depletion significantly suppressed the expression of *broad* and *HR3* in the fat bodies from sixth-instar larvae at 96 h PE (Additional file 2: Fig. S6D, G). Lys1 depletion significantly suppressed the expression of *broad* and *HR3* in the fat bodies from sixth-instar larvae at 72 h PE, whereas CD209 depletion inhibited *HR3* expression. Sixth-instar larvae injected with *L. plantarum* or fed with *L. plantarum* showed a significantly lower expression of *broad* and *HR3* at 96 h PE (Additional file 2: Fig. S6E, F, H, I). The decreased expression of *broad* and *HR3* reflects the suppression of 20E signaling, consistent with decrease in 20E titers and delayed pupariation upon CD209 or Lys1 depletion, as well as the translocation of *L. plantarum* from the midgut to hemocoel.

### Administration of 20E partially rescues CD209 or Lys1-depleted phenotypes, as well as the phenotypes caused by the translocation of *Lactobacillus* from the midgut to hemocoel

Because CD209 or Lys1 depletion, as well as the translocation of *Lactobacillus* from the midgut to hemocoel, lowered 20E titers and delayed pupariation, we wondered whether administration of 20E would rescue these phenotypes. Larvae pretreated with ds*CD209* or ds*Lys1* were randomly divided into two groups, which exhibited no obvious differences in the duration of fourth-instar and fifth-instar. 20E administration was committed at 72 h PE of sixth-instar and accelerated pupariation in CD209 and Lys1-depleted larvae for an average of ~ 3 and 10 h, respectively (Additional file 2: Fig. S7A, B). Furthermore, administration of 20E accelerated the pupariation of *L. plantarum*-injected and *L. plantarum*-fed larvae for an average of ~ 4 and 6 h, respectively (Additional file 2: Fig. S7C, D). Therefore, the delayed pupariation caused by CD209 or Lys1 depletion, as well as the translocation of *Lactobacillus* from the midgut to hemocoel, might be partially due to reduced 20E titers.

### Injection of *L. plantarum* decreases the storage of TAGs and cholesterols

To investigate why translocation of *L. plantarum* from the midgut to hemocoel lowered 20E titers, we injected bacteria into the hemocoel and performed untargeted metabolomics analyses. Partial least squares-discriminant analysis (PLS-DA) of both positive and negative modes revealed that the hemolymph samples of *L. plantarum*-injected larvae were separated clearly from the control samples (Additional file 2: Fig. S8A, B). Metabolome analysis revealed a substantial difference in the content of 433 hemolymph metabolites (Additional file 1: Table S6; Additional file 2: Fig. S8C, D). In positive ion mode, 113 potential metabolites were upregulated, whereas 112 metabolites were downregulated in the hemolymph of *L. plantarum*-injected larvae (Additional file 2: Fig. S8C). In negative ion mode, 112 and 96 metabolites were up- and downregulated, respectively (Additional file 2: Fig. S8D). Kyoto Encyclopedia of Genes and Genomes (KEGG) pathway analysis indicated that 10 differential metabolites were enriched in glycerophospholipid metabolism, including two down- and eight upregulated metabolites (Additional file 1: Table S7; Additional file 2: Fig. S8E).

**Fig. 6:**
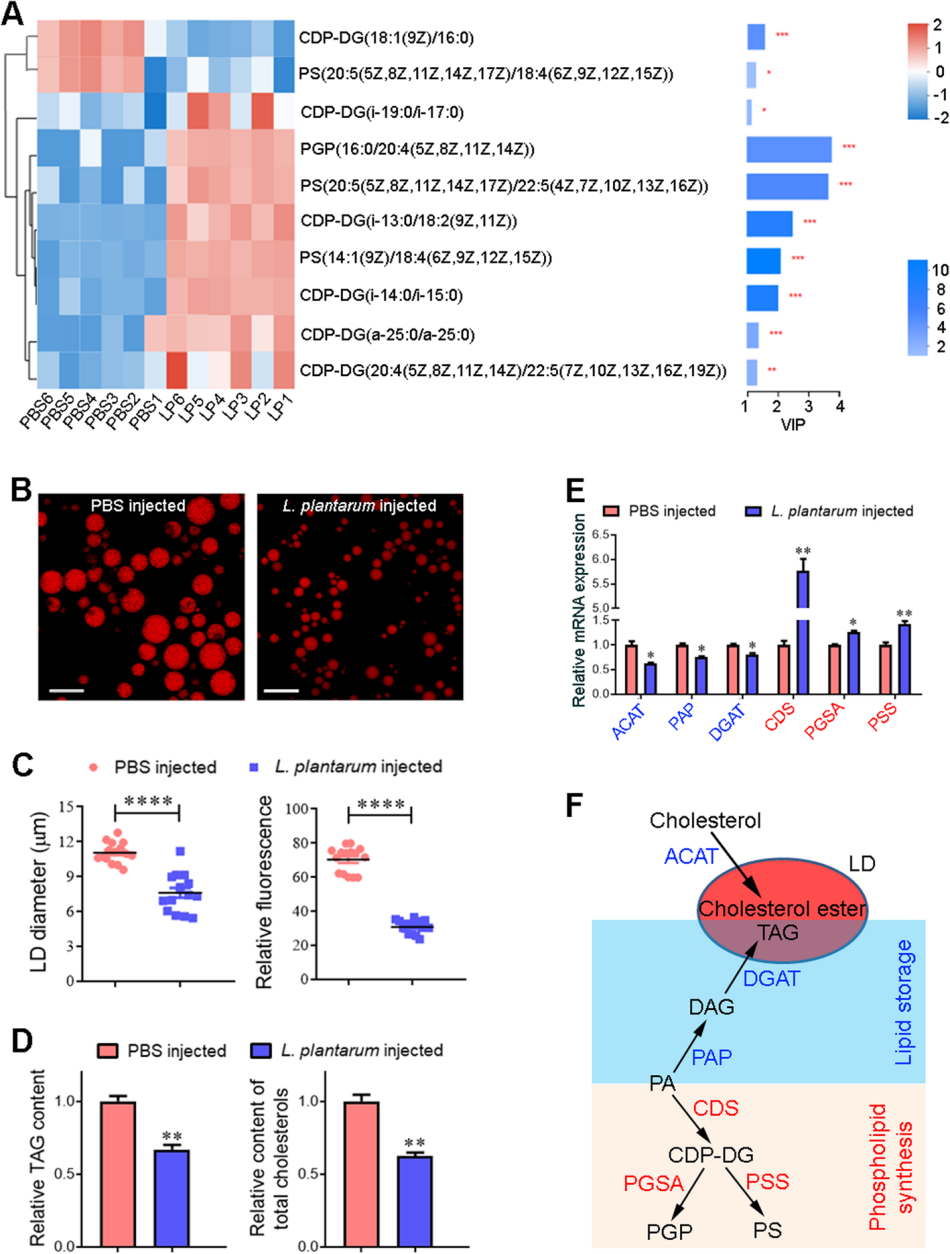
Injection of *L. plantarum* induces a shift from lipid storage to phospholipid synthesis and decreases total cholesterols. **A** Heatmap showing that most of the metabolites involved in phospholipid synthesis were increased. Hemolymph samples were collected from sixth-instar larvae at 24 h post-*L. plantarum* (LP) or -PBS injection. Variable importance in projection (VIP) scores for a metabolite represent the contribution of that metabolite to the difference between the two groups. The larger the score, the greater the difference between the two groups. **B** LDs were detected in the fat bodies of sixth-instar larvae at 24 h post-*L. plantarum* or -PBS injection. Nile red staining was used for detection. Scale bar = 20 μm. **C** LD diameter and fluorescence intensity were significantly lower in fat bodies of *L. plantarum*-injected larvae than that of PBS-injected controls. **D** The levels of TAGs and total cholesterols were significantly lower in *L. plantarum*-injected samples than that of controls. **E** Expression profiles of genes encoding enzymes involved in the synthesis of cholesterol esters, lipids, or phospholipids. Fat bodies were collected from *L. plantarum*-injected or PBS-injected larvae. ACAT, Acyl-CoA cholesterol acyltransferase (XM_021333888); PAP, PA phosphatase (XM_021327456); DGAT, Acyl-CoA diacylglycerol acyltransferase (XM_021328346); CDS, CDP-DG synthase (XM_049847014); PGSA, PG-P synthase (XM_021335307); PSS, PS synthase (XM_049835809). **F** Pathways for the synthesis of cholesterol esters, lipids, and phospholipids. LD, lipid droplet; PA, phosphatidic acid; DAG, diacylglycerol; TAG, triacylglycerol; CDP-DG, cytidine diphosphate-diacylglycerol; PG-P, phosphatidyl-glycerophosphate; PS, phosphatidylserine. Statistical differences were analyzed using Student’s *t* test (*p < 0.05, **p < 0.01, ***p < 0.001, and ****p < 0.0001)

All 10 differential metabolites, including six cytidine diphosphate-diacylglycerols (CDP-DGs), one phosphatidyl-glycerophosphate (PGP), and three phosphatidylserines (PSs), were involved in phospholipid synthesis [34, 35]. Because these metabolites were mostly increased (Fig. 6A), we inferred that *L. plantarum* challenge stimulated phospholipid synthesis. Phosphatidic acid (PA) and the enzymes that control its utilization regulate the balance between lipid storage and phospholipid production [36]. Here, we evaluated fat body lipid content by measuring the relative size and density of LDs. Both the size and fluorescence intensity of LDs decreased dramatically after *L. plantarum* challenge (Fig. 6B, C), indicating a shift in lipid homeostasis toward energy consumption. The levels of TAGs and cholesterols (mostly in the form of cholesterol esters stored in LDs) [37] decreased significantly after *L. plantarum* challenge (Fig. 6D).

The expression of acyl-CoA cholesterol acyltransferase (*ACAT*), responsible for the conversion of cholesterol into cholesterol ester [38], decreased after *L. plantarum* challenge (Fig. 6E, F). In addition, the expression of PA phosphatase (*PAP*) and acyl-CoA diacylglycerol acyltransferase (*DGAT*), which participate in TAG storage [39, 40], decreased. By contrast, transcript abundance of CDP-DG synthase (*CDS*), PG-P synthase (*PGSA*), and PS synthase (*PSS*), which are involved in phospholipid synthesis [34, 35], increased after *L. plantarum* challenge (Fig. 6E, F). These results indicated that *L. plantarum* challenge induced a shift from lipid storage to phospholipid synthesis and decreased cholesterol esters stored in LDs.

### Lysine-type PGN contributes to the reduced storage of TAGs and cholesterols, which is evolutionarily conserved

**Fig. 7:**
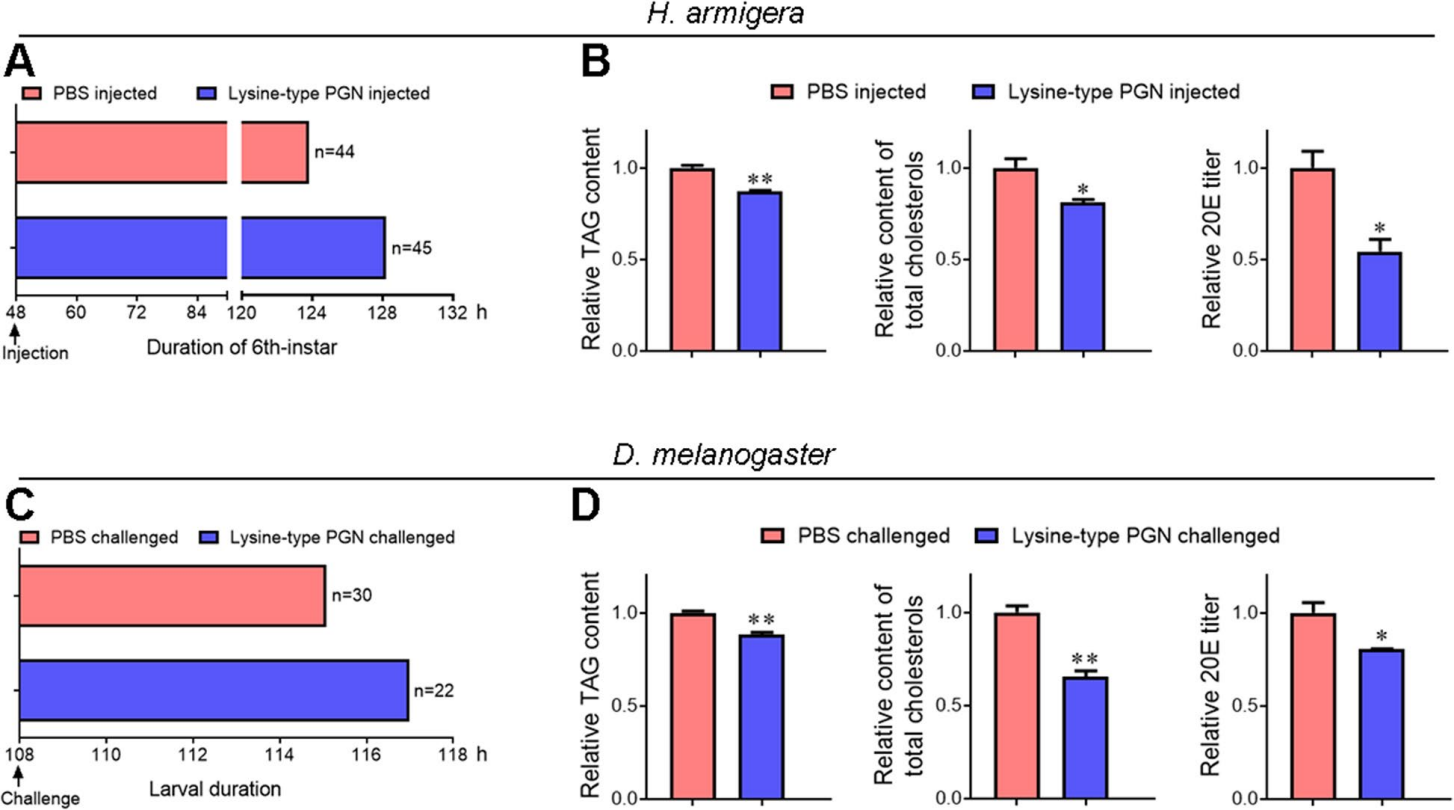
Challenge by Lysine-type PGN results in delayed pupariation and decreased levels of TAGs, cholesterols, and 20E. **A**, **C** Challenge by Lysine-type PGN delays pupariation in *H. armigera* (**A**) and *D. melanogaster* (**C**). **B**, **D** Challenge by Lysine-type PGN reduced the levels of TAGs, cholesterols, and 20E in *H. armigera* (**B**) and *D. melanogaster* (**D**). Statistical differences were analyzed using Student’s *t* test (*p < 0.05 and **p < 0.01)

Studies have indicated that PGN fragments elicit a “malaise syndrome” associated with delayed metamorphosis of *Manduca sexta* [41]. Therefore, we hypothesized that delayed pupariation and decreased storage of TAGs and cholesterols after *L. plantarum* challenge might be due to bacteria-derived PGN. Lysine-type PGN is present on the cell wall of gram-positive bacteria [42], such as *Lactobacillus*. We challenged *H. armigera* and *Drosophila melanogaster* larvae with Lysine-type PGN, followed by measuring the duration of larval instar and the levels of TAGs, cholesterols, and 20E. Challenge by Lysine-type PGN delayed pupariation in *H. armigera* and *D. melanogaster* for an average of ~ 4 and 2 h, respectively (Fig. 7A, C). Challenge with Lysine-type PGN significantly decreased the levels of TAGs, cholesterols, and 20E in both *H. armigera* and *D. melanogaster* larvae (Fig. 7B, D). Therefore, the reduced storage of lipids and cholesterol esters in *L. plantarum*-injected *H. armigera* was at least partially due to bacteria-derived Lysine-type PGN. A similar phenomenon has been observed in *D. melanogaster*, suggesting that PGN (Lysine-type)-triggered lipid and cholesterol reduction is evolutionarily conserved.

## Discussion

**Fig. 8:**
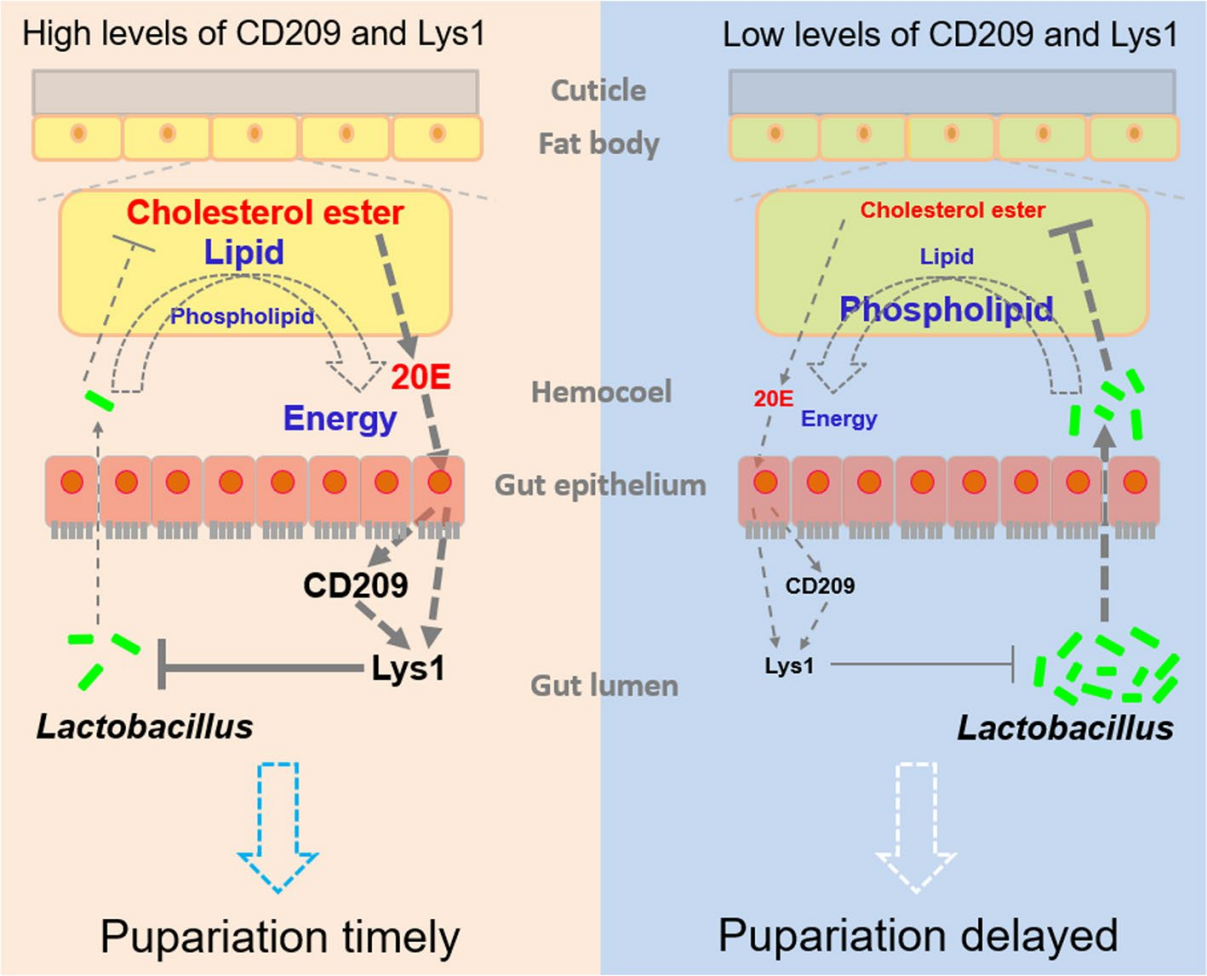
Model showing CTL − Lys immune function regulates gut homeostasis and controls insect metamorphosis. CD209 and its downstream Lys1 maintain high expression levels in the midgut during the wandering stage due to the high 20E titers. Lys1 suppresses the proliferation of *Lactobacillus* in the midgut, resulting in a low abundance of *Lactobacillus* (and the associated PGN) in the hemocoel. In this condition, fat body cells are mainly involved in the storage of lipids and cholesterol esters in LDs, thus providing sufficient energy and 20E titers for timely pupariation. However, low expression levels of CD209 and Lys1 increase the *Lactobacillus* load in the midgut. The increased loads of *Lactobacillus* (and PGN) subsequently translocate into the hemocoel and trigger a shift from lipid storage to phospholipid synthesis. Moreover, cholesterol esters stored in LDs decrease. This leads to insufficient energy and 20E titers, thereby delaying pupariation. The low 20E titers, in turn, fail to induce high levels of CD209 and Lys1 expression in the midgut

The immune system shapes gut bacterial community composition and abundance [9, 21], and some gut symbiotic bacteria are involved in modulating host development in the midgut [3, 11]. Here, we demonstrated that the immune system modulates insect metamorphosis by shaping the gut bacteria and restricting opportunistic infections. As summarized in Fig. 8, we elucidated the essential role of a CD209 (CTL) − Lys1 (Lys) immune function in maintaining gut homeostasis, thereby minimizing the risk of opportunistic infections during complete metamorphosis. We showed that opportunistic infections caused by gut *Lactobacillus* result in insufficient energy and 20E, thus delaying insect metamorphosis. Moreover, 20E elevates the expression of CD209 and Lys1, which in turn maintain high 20E titers by suppressing *Lactobacillus* production in the midgut and hemolymph during the wandering stage. Our results reveal a positive feedback loop of endocrine–immune interactions mediated by *Lactobacillus*, highlighting the significance of gut homeostasis and local–systemic communication during complete metamorphosis.

### Endogenous 20E-mediated increase in immunity contributes to effective gut sterilization, thereby minimizing the risk of opportunistic infections during complete metamorphosis

Consistent with reports on species, such as mosquitoes [43], our results indicated that although the diversity of gut bacteria increased, their load largely decreased when larvae entered the wandering stage. *Lactobacillus* accounted for the gut bacteria with the largest decrease. By contrast, the total bacterial load in the hemolymph increased significantly during metamorphosis, possibly from the metamorphosing midgut, as did *Lactobacillus*. Because the peritrophic membrane, which lines the midgut lumen and prevents gut microbial diffusion, is purged at the onset of metamorphosis [8, 44], we believe that the fewer gut bacteria there are, the lower the risk of opportunistic infections. The transcript abundance of most of the PRRs and antibacterial effectors increased in the metamorphosing midgut, as reported in *Bombyx mori* [45]. Endogenous 20E titers contributed to the high level of CD209 and Lys1, and possibly other immunity-related genes, because they were upregulated in the metamorphosing midgut. Considering that loss of immunity-related genes, such as antibacterial effectors, leads to an increased load of gut microbiota [9], 20E-mediated increase in immunity might be an effective gut sterilization mechanism during complete metamorphosis.

### CD209 and its downstream Lys1 restrict *Lactobacillus* during metamorphosis

Lys accumulates in the midgut epithelium and is released into the gut lumen after the larval epithelium has been sloughed off during metamorphosis [46]. In *Drosophila*, Lys loss results in an increased load of gut microbiota [9]. A similar result was observed in this study, with Lys1 depletion leading to an increased load of gut *Lactobacillus*. We further showed that Lys1 directly targets *L. plantarum* to suppress its proliferation, which can be explained by the fact that Lys can cleave PGN on the cell wall of gram-positive bacteria [47]. However, we cannot rule out the possibility that Lys1 contributes to gut immunity by modulating immune signaling, because Lys also cleaves PGN shed from gut microbiota [48]. Similar with the function of Lys1 in restricting gut *Lactobacillus* during metamorphosis, as a CTL, CD209 was suggested to act upstream of Lys1. Although many CTLs are known to regulate the expression of antibacterial effectors [15, 49, 50], the detailed regulatory mechanisms are unclear. Since injection of dsRNA results in systemic rather than gut specific knockdown of the target gene, the effect of downregulation of CD209 or Lys1 in other tissues cannot be ruled out. However, considering the predominant expression of CD209 and Lys1 in the metamorphosing midgut, our explanation is reasonable.

### Increased load of *Lactobacillus* and its associated PGN translocate from the midgut to hemocoel, delaying metamorphosis in CD209- or Lys1-depleted larvae

*L. plantarum*, as a commensal bacterium in the gut, exerts its benefits by promoting *Drosophila* systemic growth [3]. Similar to *E. faecalis* in *M. sexta* [14], however, *L. plantarum* in *H. armigera* completes its commensal-to-pathogen switch after translocation from the midgut to hemocoel. Opportunistic infections caused by gut *L. plantarum* did not induce sepsis resulting in *H. armigera* death, but elicited delayed metamorphosis of the final larval instar. Injection of gut *E. mundtii* into the hemocoel induced a similar phenotype in *H. armigera* [15], suggesting that it is not bacteria-specific and a common mechanism may be involved. A “malaise syndrome” associated with delayed metamorphosis was characterized in *M. sexta* and could be elicited by PGN [41]. Given that PGN is present on the cell wall of bacteria and is shed during bacterial growth and maturation [42], we suggest that the delayed metamorphosis caused by opportunistic infections is triggered by the PGN derived from gut bacteria that invade the hemocoel. Lysine-type PGNs are derived from gram-positive bacteria [42], such as *L. plantarum* and *E. mundtii*. Delayed pupariation was observed in both *H. armigera* and *D. melanogaster* after Lysine-type PGN challenge, supporting this hypothesis. Gut microbiota-shed PGN could translocate from the gut lumen into systemic circulation [51]. Therefore, it was suggested that the Lysine-type PGN derived from both gut and hemolymph *Lactobacillus* contributed to delayed metamorphosis in CD209- or Lys1-depleted larvae.

### High abundance of *Lactobacillus* and its associated Lysine-type PGN in hemocoel decreases lipids and cholesterol esters stored in LDs, resulting in insufficient energy and 20E available for metamorphosis

Fighting infection is an energy-consuming process [16], as is committing metamorphosis [52], so there may be a competition for energy between the two. Metabolism, such as lipid metabolism, needs to fulfill the energy requirements of an organism [53]. Opportunistic infections caused by gut *L. plantarum* and its associated Lysine-type PGN perturb lipid homeostasis, leading to a reduction in fat body lipids, which are the primary source of energy during metamorphosis [52]. This may be one of the causes why opportunistic infections delay metamorphosis. Shifting lipid metabolism from TAG storage to membrane phospholipid synthesis also occurs in *Drosophila* upon activation of toll signaling. This shift favors endoplasmic reticulum expansion and sustains the immediate demand for AMP secretion during infection [54]. Because fat body cells are major sites for energy metabolism and innate immunity [55], their coordination during metamorphosis is of great interest.

Ecdysone, produced when needed from cholesterol esters stored in LDs [18, 37], is essential for triggering insect metamorphosis [19]. Interestingly, we found that the level of cholesterol esters decreased with the decrease of LD size in *L. plantarum*-injected larvae. The level of ecdysone correspondingly decreased. Therefore, insufficient cholesterol mobilization and ecdysone production may be another cause of delayed metamorphosis due to opportunistic infections. This was confirmed by the administration of 20E, which partially rescued pupariation in *L. plantarum*-injected or fed larvae. The expression of Halloween genes involved in ecdysteroid biosynthesis are suppressed in *E. mundtii*-injected larvae [15], suggesting that endocrinological changes caused by opportunistic infections are multilayered. Given that 20E suppresses TAG storage [56], low 20E levels may facilitate lipid storage to compensate for energy expenditure during the fight against infections.

Lysine-type PGN induced storage defects in lipids and cholesterol esters, suggesting that delayed metamorphosis due to energy and ecdysone deficiency is applicable to opportunistic infections caused by gram-positive bacteria. Similar phenomena, such as delayed pupariation and decreased storage of lipids and cholesterol esters, have been observed in *Drosophila* larvae challenged by Lysine-type PGN, suggesting that this is a common mechanism.

## Conclusions

Opportunistic infections caused by gut symbiotic bacteria occur frequently [57, 58], affecting mammal or insect development through mechanisms that are obscure. Here, we have shown that opportunistic infections caused by gut *Lactobacillus* lead to a reduction in storage of lipids and cholesterols, thereby reducing the availability of energy and ecdysone for insect pupariation. CD209 and its downstream Lys1, induced by ecdysone, contribute to gut homeostasis, thereby minimizing the risk of opportunistic infections (Fig. 8). Therefore, the endocrine system controls the risk of opportunistic infections by recruiting gut immunity, which in turn ensures normal endocrine function and timely metamorphosis.

## Methods

### Insect rearing, antibiotic treatment, and PGN challenge

*H. armigera* larvae were maintained in an insectarium under the photoperiod of 14 h light/10 h dark at 27 ± 1°C. The larvae were fed on an artificial diet comprising soybean powder, wheat germ, compound vitamins, and mineral salt. Axenic *H. armigera* larvae were obtained by constantly feeding on a diet supplemented with gentamicin (15 μg/mL), penicillin (10 units/mL), and streptomycin (10 μg/mL) from the third instar. The midguts were dissected from sixth-instar larvae at 0 h PE, reared on a diet with or without antibiotics. The efficacy of cultivable bacterial elimination was confirmed by plating gut homogenates onto LB agar plates. PCR analysis against gut homogenates was conducted using universal 16S rRNA gene primers 16S-F and 16S-R (Additional file 1: Table S8). For PGN challenge, sixth-instar larvae at 48 h PE were injected with 5 μL of Lysine-type PGN (500 ng/μL, KALANG, Shanghai, China).

*D. melanogaster* were raised on a standard cornmeal/yeast diet under the photoperiod of 8 h light/16 h dark at 25 ± 1°C. *Drosophila* late-third instar larvae (~ 108 h after egg lay) were collected and rinsed in sterile PBS. The larvae were punctured laterally with a needle dipped in Lysine-type PGN or PBS. The punctures were performed in mineral oil, where the punctured larvae were kept for another 10 min before transfer to fresh food, as described [54].

### Sequencing and analysis of bacterial 16S rRNA gene from the midgut

Midguts were collected from sixth-instar larvae at 24 and 72 h PE. Before the midguts were dissected, larvae were surface-sterilized with 75% ethanol. Five independent biological replicates were included. A total of 20 midguts from each stage were pooled as a replicate and used for metagenomic DNA extraction. The V3 and V4 hypervariable regions of the 16S rRNA gene were amplified with primers 338F and 806R (Additional file 1: Table S8). The PCR amplicon pools were prepared for sequencing on an Illumina MiSeq platform (San Diego, USA) by Majorbio Bio-Pharm Technology Co., Ltd. (Shanghai, China). The raw sequencing reads were demultiplexed, quality-filtered by Trimmomatic, and merged using FLASH. OTUs with 97% similarity cut-off were clustered using UPARSE (http://drive5.com/uparse/). The taxonomy of each OTU representative sequence was analyzed by RDP Classifier (http://rdp.cme.msu.edu/) against the SILVA database (http://www.arb-silva.de) using a confidence threshold of 0.7.

### Isolation, characterization, inoculation, and colonization of *L. plantarum*

To isolate bacteria from the midgut, sixth-instar larvae at 24 h PE were surface-sterilized in 75% ethanol and rinsed in sterile PBS. The midguts were dissected, homogenized, and diluted (1:1000) in sterile PBS. The diluted homogenates were spread on MRS (Solarbio, Beijing, China) agar plates. The plates were incubated at 37°C for 24 − 48 h. Morphologically distinct colonies were selected for genomic DNA extraction using a bacterial DNA kit (Omega, GA, USA). DNA fragments were amplified using universal 16S rRNA gene primers 27F and 1492R (Additional file 1: Table S8), followed by sequencing. The closest *L. plantarum* matching the 16S rRNA gene sequence (99% identity) was obtained after BLAST search (https://blast.ncbi.nlm.nih.gov/Blast.cgi) against the 16S rRNA database.

*L. plantarum* in mid-logarithmic phase was harvested, washed twice, and resuspended in PBS at 2 × 10^8^ cells/mL. Each sixth-instar larva at 48 h PE was injected with 5 μL of bacterial suspension. *L. plantarum* was introduced into sixth-instar larvae at 0 h PE by feeding on artificial diet, supplemented with the bacterial suspension. An equal amount of sterile PBS was used as a control. Midguts, hemolymph, and fat bodies from the bacteria-injected or -fed larvae were collected for further assays.

### RNAi

To produce ds*CD209* and ds*Lys1*, the fragments of these two genes were amplified using the primers listed in Table S8 (Additional file 1). The ds*CD209* and ds*Lys1* were synthesized with their respective PCR products as templates using a MEGAscript kit (Ambion, Austin, TX, USA). The ds*GFP* was synthesized as a control. The dsRNAs were eluted from the purification column with nuclease-free water and concentrated to 2.0 μg/μL. Each fourth-instar larva at 6 − 12 h PE was injected with 5 μL of ds*CD209*, ds*Lys1*, or ds*GFP*. The midguts, hemolymph, and fat bodies were collected from the larvae after dsRNA injection for analysis.

### RNA-seq analysis

Total RNA was extracted from the midguts of sixth-instar larvae at 24 h PE (feeding; MgF) and 72 h PE (wandering; MgW), and those at 72 h PE pretreated with ds*CD209* or ds*GFP* using TRIzol (Invitrogen, Carlsbad, CA, USA). The cDNA libraries (MgF-1, -2, -3, -4; MgW-1, -2, -3, -4; ds*CD209*-1, -2, -3; ds*GFP*-1, -2, and -3) were constructed as described [59]. Briefly, mRNAs were isolated using oligo(dT) beads and then fragmented. Double-stranded cDNAs were synthesized using EasyScript cDNA synthesis SuperMix (TransGen Bio-tech, Beijing, China), followed by sequencing on the Illumina Novaseq 6000 platform (San Diego, USA) at Majorbio Bio-Pharm Technology Co., Ltd. Clean reads were obtained after filtration of raw reads and aligned to the *H. armigera* genome (https://www.ncbi.nlm.nih.gov/genome/13316?genome_assembly_id=319039) using TopHat software (http://tophat.cbcb.umd.edu/). Mapped reads were assembled and all unigenes were run in BLASTx against the non-redundant database. DEGs in the libraries (MgW vs. MgF; ds*CD209* vs. ds*GFP*) were determined using a fold cut-off change value ≥ 2 and adjusted *p*-value < 0.05.

### Measurement and injection of 20E

We determined 20E titers using the double antibody sandwich method with an insect 20E ELISA kit (Kmaels Biotechnology, Shanghai, China), as described [60]. Hemolymph was collected from sixth-instar larvae at 72 and 96 h PE treated with dsRNA or *L. plantarum*. Hemolymph from five larvae was pooled as a replicate, and three replicates in each treatment were obtained for 20E measurement. For *Drosophila*, larvae were collected at 3 h post-PGN challenge, homogenized, and centrifuged. The supernatants were used for assays. The samples were added to the microwells precoated with 20E antibodies. Subsequently, horseradish peroxidase-labeled detection antibodies were added to the wells. After thorough washing was complete, 3,3′,5,5′-tetramethylbenzidine was used for color development. Absorbance was measured at 450 nm, and 20E titers were calculated from the standard curve.

To determine gene expression, each sixth-instar larva at 48 h PE was injected with 20E (ABMole, Houston, TX, USA) at 200 ng/5 μL, which was close to the amount of endogenous 20E during the wandering stage [26]. An equal amount of dimethyl sulfoxide (DMSO), used for dissolving 20E, was injected as a solvent control. Three biological replicates were included, with each replicate comprising four larvae. Midguts were collected for total RNA or protein extraction at 12 and 24 h post-injection.

### Quantification of total bacteria and *Lactobacillus* in the midgut and hemolymph

Genomic DNA from the midguts and hemolymph of sixth-instar larvae at 24 or 72 h PE was extracted using a genomic DNA isolation kit (Omega). Genomic DNA was extracted from the midguts and hemolymph of sixth-instar larvae at 72 h PE after treatment with ds*CD209*, ds*Lys1*, or ds*GFP*. Moreover, genomic DNA was isolated from the midguts and hemolymph of sixth-instar larvae at 24 and 72 h PE fed with a diet containing *L. plantarum* or PBS. The qPCR analysis was performed on genomic DNA using universal 16S rRNA gene primers 16S-F and 16S-R or *Lactobacillus*-specific 16S rRNA primers (Additional file 1: Table S8). The qPCR was performed using TransStart Tip Green qPCR SuperMix (TransGen Bio-tech). *H. armigera* β-actin gene was used as an endogenous control.

### Recombinant protein generation, bacterial-binding assay, and antiserum preparation

The DNA fragment encoding the mature peptide of CD209 or Lys1 was cloned into pET32a plasmids, which were transformed into *Escherichia coli* BL21 (DE3) competent cells. After addition of 0.3 mM isopropyl β-D-1-thiogalactopyranoside (IPTG) at 16°C overnight, rCD209 was induced in a soluble form, whereas rLys1 was present in inclusion bodies. The inclusion bodies were denatured and renatured, as described [61]. The recombinant proteins were purified using High-Affinity Ni–NTA Resin (Novagen, WI, USA), dialyzed against PBS, and concentrated to desired concentrations. The rTrx was prepared simultaneously and used as control.

*L. plantarum* (2 × 10^8^ cells/mL) was incubated with rCD209 (1.0 mg/mL) in the presence or absence of 10 mM CaCl_2_ for 30 min. The bacterial cells were pelleted, washed, and eluted with 7% SDS. The rTrx was used as negative control. The samples were analyzed by western blotting.

After homogenization of rCD209 (200 μg) with complete Freund’s adjuvant, the mixture was hypodermically injected into the back of a rabbit. After 3 weeks, the same amount of recombinant protein, homogenized with incomplete Freund’s adjuvant, was subcutaneously injected. The antiserum against CD209 was prepared after a booster injection of 500 μg antigen 2 weeks later.

### Western blotting

Total proteins isolated from various tissues, including the midgut treated with 20E or RNAi, were subjected to western blot analyses. Purified rCD209 or rTrx were loaded as controls. Equal aliquots of protein samples (100 μg/lane) were resolved using 12.5% SDS-PAGE and transferred to nitrocellulose membranes (Millipore, Darmstadt, Germany). The membranes were blocked with 5% skim milk and incubated with the antiserum against rCD209 at a 1:500 dilution. An antibody against β-actin (ABclonal, Cat. AC026) was used as a loading control. Protein samples from bacterial washing and elution were subjected to western blot analyses using anti-His antibody (ABclonal, Cat. AE003). Dosage analyses were performed using ImageJ software (Bethesda, MD, USA), based on three biological replicates.

### Analysis of the antibacterial activity of Lys1

To analyze the antibacterial activity of Lys1, rLys1 was incubated with enterokinase (Beyotime Bio-tech, Shanghai, China) to remove Trx (His-tagged). A total of 90 μL of Lys1 (25 μM) was mixed with 10 μL of bacterial suspension at 25°C for 1 h. The mixture was plated on MRS agar plates and cultured at 37°C for 24 h. The number of colonies on each plate was recorded. The same concentration of bovine serum albumin (BSA) was used as a control. Three replicates were used.

### RT-qPCR

Total RNA was extracted from various tissues, including the midgut treated with 20E or RNAi, using TRIzol Reagent (Invitrogen). Total RNA was isolated from fat bodies of sixth-instar larvae at 72 and 96 h PE after either dsRNA injection or *L. plantarum* treatment. First-strand cDNA was synthesized using EasyScript cDNA synthesis SuperMix (TransGen Bio-tech). RT-qPCR analysis was performed using TransStart Tip Green qPCR SuperMix (TransGen Bio-tech). The housekeeping gene β-actin was used as an endogenous control. Primers used for RT-qPCR analysis are listed in Table S8 (Additional file 1).

### Untargeted metabolomics analysis

Hemolymph samples (100 μL) from sixth-instar larvae at 24 h post-*L. plantarum* or -PBS injection were mixed with 400 μL extraction solution (acetonitrile:methanol = 1:1) containing 0.02 mg/mL internal standard (L-2-chlorophenylalanine). After vortexing for 30 s and low-temperature ultrasonic extraction for 30 min was complete, the samples were kept at − 20°C for 30 min to precipitate the proteins and centrifuged for 15 min at 13,000 × *g*. The supernatants were collected and blow-dried under nitrogen. The samples were resolubilized with 100 μL solution (acetonitrile:water = 1:1) and centrifuged at 13,000 × *g*. The supernatants were obtained for LC–MS/MS analysis.

The metabolite extracts from hemolymph samples were analyzed using a UPLC-Q-Exactive mass spectrometer (Thermo Fisher Scientific, MA, USA). Chromatographic separation was conducted using an ACQUITY HSS T3 column (Waters, Milford, USA). The mobile phases consisted of 0.1% formic acid in water:acetonitrile (95:5; solvent A) and 0.1% formic acid in water:acetonitrile:isopropanol (5:47.5:47.5; solvent B). Raw data were processed with Progenesis QI software (Waters). Internal standard peaks and known false positive peaks were removed. The metabolites were searched against the HMDB database (http://www.hmdb.ca/). The R package “ropls” (version 1.6.2) was used to perform PLS-DA. Metabolites with VIP > 1 and *p* < 0.05 were considered significantly different metabolites. Differential metabolites between two groups were mapped into their biochemical pathways through metabolic enrichment based on the KEGG database (http://www.genome.jp/kegg/).

### LD staining

Fat bodies were collected at 24 h post-injection of *L. plantarum* or PBS, incubated in Nile red solution (20% glycerol in PBS, with 1:500 dilution of 10% Nile red in DMSO) for 20 min, and imaged under a laser-scanning confocal microscope (Leica, Germany). LD diameter and fluorescence intensity were determined using ImageJ software. Each group consisted of 15 average LD diameters and fluorescence intensities, each of which was based on one image.

### TAG and cholesterol assays

Fat bodies were collected at 24 h post-*L. plantarum*, -PGN (Lysine-type), or -PBS injection to determine TAG and cholesterol levels, as described [62]. Fat bodies were homogenized in isopropanol and centrifuged at 10,000 × *g* for 10 min. The supernatants were used for TAG and cholesterol quantification using a triglyceride colorimetric assay kit (Elabscience, Wuhan, China) and total cholesterol colorimetric assay kit (Elabscience), respectively. For *Drosophila*, larvae were collected at 3 h post-PGN challenge, homogenized, and centrifuged, following which the supernatants were used for assays.

### Measurement of developmental timing

To determine the mean duration of the fourth-, fifth-, and sixth-instars, we recorded each individual every 1 h from the beginning of each treatment until pupariation. The duration measurement was based on 23 ds*CD209*- and 24 ds*GFP*-injected larvae, as well as 28 ds*Lys1*- and 34 ds*GFP*-injected larvae. A total of 28 *L. plantarum*- or PBS-injected larvae, 36 *L. plantarum*-fed larvae, and 41 PBS-fed larvae were used for the assays. A total of 45 PGN (Lysine-type)- and 44 PBS-injected larvae were assayed. For *Drosophila*, 22 PGN (Lysine-type)- and 30 PBS-challenged larvae were analyzed. The mean duration of larval stages was determined.

To determine whether administration of 20E rescued delayed pupariation, sixth-instar larvae pre-treated with ds*CD209* or ds*Lys1* were divided into two groups. Sixth-instar larvae injected or fed with *L. plantarum* were divided into two groups. One group was injected with 5 μL of 20E (500 ng/μL) per larva, whereas the other group was injected with an equivalent amount of DMSO as solvent control. Both injections were committed at 72 h PE of sixth-instar. In the ds*CD209*-injected group, 34 20E- and 29 DMSO-injected larvae were analyzed. In the ds*Lys1*-injected group, 33 20E- and 30 DMSO-injected larvae were analyzed. In the *L. plantarum*-injected group, 32 20E- and 30 DMSO-injected larvae were used for calculating the mean of the sixth-instar. In the *L. plantarum*-fed group, 35 20E- and 34 DMSO-injected larvae were analyzed. Each analysis was conducted twice.

### Statistical analyses

Statistical analysis was performed using GraphPad Prism 8 (GraphPad Inc., La Jolla, CA, USA). The Mann–Whitney *U* test was used to evaluate differences in bacterial composition between the two groups. Unless otherwise indicated, Student’s *t* test was used to determine differences between two groups. One-way analysis of variance (ANOVA) coupled with Tukey’s multiple comparison test was used to evaluate differences among multiple groups. A value of *p* < 0.05 was considered statistically significant.

### Supplementary Information

**Additional file 1:** **Table S1. **Midgut OTU taxonomy from sixth-instar larvae of the feeding stage (MgF) and the wandering stage (MgW). **Table S2. **DEGs between the midguts from sixth-instar larvae of the feeding stage (MgF) and the wandering stage (MgW). **Table S3.** Immunity-related DEGs between the midguts from sixth-instar larvae of the feeding stage (MgF) and the wandering stage (MgW). **Table S4.** DEGs between the midguts from sixth-instar larvae treated with ds*CD209* and ds*GFP*. **Table S5. **Immunity-related DEGs between the midguts of sixth-instar larvae treated with ds*CD209* and ds*GFP*. **Table S6. **Differential metabolites between the hemolymph samples from sixth-instar larvae injected with *L. plantarum* (LP) and PBS. **Table S7. **Differential metabolites enriched in glycerophospholipid metabolism between the hemolymph samples from sixth-instar larvae injected with *L. plantarum* (LP) and PBS. **Table S8. **Primers used in this study.

**Additional file 2:** **Figure S1.** Quantification of total bacteria in the midgut (**A**) and hemolymph (**B**) in the feeding and wandering stages. **Figure S2. **20E induces the expression of CD209 and Lys1 in the midgut. **Figure S3. **Binding of CD209 to *L. plantarum* independent of Ca^2+^. **Figure S4. **Conserved protein sequence of *H. armigera* Lys1 with C-type Lys. **Figure S5. **Efficiency of gut bacterial elimination. **Figure S6. **Suppression in 20E signaling in CD209- or Lys1-depleted larvae, as well as in *L. plantarum*-injected or *L. plantarum*-fed larvae. **Figure S7. **20E treatment shortens the duration of sixth-instar in CD209- or Lys1-depleted larvae, as well as in *L. plantarum*-injected or *L. plantarum*-fed larvae. **Figure S8. **Metabolomics analysis of hemolymph samples from *H. armigera* larvae injected with *L. plantarum* (LP) or PBS.

**Additional file 3.** Original images of western blot and PCR gel.

## Data Availability

The 16S rRNA gene and RNA-sequencing data have been deposited in the NCBI Sequence Read Archive under the accession number PRJNA1051296 [63], PRJNA1042681 [64], and PRJNA1043398 [65]. Metabolomics raw data have been deposited in Mendeley Data [66].

## Abbreviations

CTL: C-type lectin
Lys: Lysozyme
CD209: CD209 antigen-like protein 2
rTrx: Recombinant thioredoxin
AMP: Antimicrobial peptide
PRR: Pattern recognition receptor
PGRP: Peptidoglycan recognition protein
PGN: Peptidoglycan
GFP: Green fluorescent protein
CecD: Cecropin D
HR3: Hormone receptor 3
CRD: Carbohydrate recognition domain
DEG: Differentially expressed gene
20E: 20-Hydroxyecdysone
PGP: Phosphatidyl-glycerophosphate
PS: Phosphatidylserine
PA: Phosphatidic acid
ACAT: Acyl-CoA cholesterol acyltransferase
PAP: PA phosphatase
DGAT: Acyl-CoA diacylglycerol acyltransferase
CDS: CDP-DG synthase
PGSA: PG-P synthase
PSS: PS synthase
TAG: Triacylglycerol
CDP-DG: Cytidine diphosphate-diacylglycerol
LD: Lipid droplet
PE: Post-ecdysis
OTU: Operational taxonomic unit
PCoA: Principal coordinate analysis
PLS-DA: Partial least squares-discriminant analysis
ANOVA: Analysis of variance
KEGG: Kyoto Encyclopedia of Genes and Genomes
qPCR: Quantitative PCR
DMSO: Dimethyl sulfoxide
IPTG: Isopropyl β-D-1-thiogalactopyranoside
BSA: Bovine serum albumin
MRS: DeMan Rogosa Sharpe
LB: Luria-Bertani
MgF: Midguts of feeding
MgW: Midguts of wandering

## References

[1] Moran N (1994). Adaptation and constraint in the complex life cycles of animals. Annu Rev Ecol Syst.

[2] Karamipour N, Fathipour Y, Mehrabadi M (2021). Removal of gut symbiotic bacteria negatively affects life history traits of the shield bug. Graphosoma lineatum Ecol Evol.

[3] Storelli G, Defaye A, Erkosar B, Hols P, Royet J, Leulier F (2011). *Lactobacillus plantarum* promotes *Drosophila* systemic growth by modulating hormonal signals through TOR-dependent nutrient sensing. Cell Metab.

[4] Johnston PR, Rolff J (2015). Host and symbiont jointly control gut microbiota during complete metamorphosis. PLoS Pathog.

[5] Stoll S, Feldhaar H, Fraunholz MJ, Gross R (2010). Bacteriocyte dynamics during development of a holometabolous insect, the carpenter ant *Camponotus floridanus*. BMC Microbiol.

[6] Akira S, Uematsu S, Takeuchi O (2006). Pathogen recognition and innate immunity. Cell.

[7] Lin Z, Wang JL, Cheng Y, Wang JX, Zou Z (2020). Pattern recognition receptors from lepidopteran insects and their biological functions. Dev Comp Immunol.

[8] Song X, Wang M, Dong L, Zhu H, Wang J (2018). PGRP-LD mediates *A. stephensi* vector competency by regulating homeostasis of microbiota-induced peritrophic matrix synthesis. PLoS Pathog.

[9] Marra A, Hanson MA, Kondo S, Erkosar B, Lemaitre B (2021). *Drosophila* antimicrobial peptides and lysozymes regulate gut microbiota composition and abundance. MBio.

[10] Wei G, Lai Y, Wang G, Chen H, Li F, Wang S (2017). Insect pathogenic fungus interacts with the gut microbiota to accelerate mosquito mortality. Proc Natl Acad Sci U S A.

[11] Shin SC, Kim SH, You H, Kim B, Kim AC, Lee KA (2011). *Drosophila* microbiome modulates host developmental and metabolic homeostasis via insulin signaling. Science.

[12] Hammer TJ, Moran NA (2019). Links between metamorphosis and symbiosis in holometabolous insects. Philos Trans R Soc Lond B Biol Sci.

[13] Ma M, Tu C, Luo J, Lu M, Zhang S, Xu L (2021). Metabolic and immunological effects of gut microbiota in leaf beetles at the local and systemic levels. Integr Zool.

[14] Mason KL, Stepien TA, Blum JE, Holt JF, Labbe NH, Rush JS (2011). From commensal to pathogen: translocation of *Enterococcus faecalis* from the midgut to the hemocoel of *Manduca sexta*. MBio.

[15] Wang W, Wang G, Zhuo X, Liu Y, Tang L, Liu X (2020). C-type lectin-mediated microbial homeostasis is critical for *Helicoverpa armigera* larval growth and development. PLoS Pathog.

[16] DiAngelo JR, Bland ML, Bambina S, Cherry S, Birnbaum MJ (2009). The immune response attenuates growth and nutrient storage in *Drosophila* by reducing insulin signaling. Proc Natl Acad Sci U S A.

[17] Yiu JH, Dorweiler B, Woo CW (2017). Interaction between gut microbiota and toll-like receptor: from immunity to metabolism. J Mol Med (Berl).

[18] Texada MJ, Malita A, Rewitz K (2019). Autophagy regulates steroid production by mediating cholesterol trafficking in endocrine cells. Autophagy.

[19] Di Cara F, King-Jones K (2013). How clocks and hormones act in concert to control the timing of insect development. Curr Top Dev Biol.

[20] Rus F, Flatt T, Tong M, Aggarwal K, Okuda K, Kleino A (2013). Ecdysone triggered PGRP-LC expression controls *Drosophila* innate immunity. EMBO J.

[21] Guo L, Karpac J, Tran SL, Jasper H (2014). PGRP-SC2 promotes gut immune homeostasis to limit commensal dysbiosis and extend lifespan. Cell.

[22] Loch G, Zinke I, Mori T, Carrera P, Schroer J, Takeyama H (2017). Antimicrobial peptides extend lifespan in *Drosophila*. PLoS ONE.

[23] Nunes C, Koyama T, Sucena É (2021). Co-option of immune effectors by the hormonal signalling system triggering metamorphosis in *Drosophila melanogaster*. PLoS Genet.

[24] Kriticos DJ, Ota N, Hutchison WD, Beddow J, Walsh T, Tay WT (2015). The potential distribution of invading *Helicoverpa armigera* in North America: is it just a matter of time?. PLoS ONE.

[25] Wang JL, Jiang XJ, Wang Q, Hou LJ, Xu DW, Wang JX (2007). Identification and expression profile of a putative basement membrane protein gene in the midgut of *Helicoverpa armigera*. BMC Dev Biol.

[26] Liu J, Shi GP, Zhang WQ, Zhang GR, Xu WH (2006). Cathepsin L function in insect moulting: molecular cloning and functional analysis in cotton bollworm, *Helicoverpa armigera*. Insect Mol Biol.

[27] Pang X, Xiao X, Liu Y, Zhang R, Liu J, Liu Q (2016). Mosquito C-type lectins maintain gut microbiome homeostasis. Nat Microbiol.

[28] Zhu YT, Zhang X, Wang SC, Li WW, Wang Q (2016). Antimicrobial functions of EsLecH, a C-type lectin, via JNK pathway in the Chinese mitten crab, *Eriocheir sinensis*. Dev Comp Immunol.

[29] Bi J, Ning M, Li J, Zhang P, Wang L, Xu S (2020). A C-type lectin with dual-CRD from *Tribolium castaneum* is induced in response to bacterial challenge. Pest Manag Sci.

[30] Mohamed AA, Zhang L, Dorrah MA, Elmogy M, Yousef HA, Bassal TT (2016). Molecular characterization of a c-type lysozyme from the desert locust, *Schistocerca gregaria* (Orthoptera: Acrididae). Dev Comp Immunol.

[31] Wang ZZ, Zhan LQ, Chen XX (2018). Two types of lysozymes from the whitefly *Bemisia tabaci*: Molecular characterization and functional diversification. Dev Comp Immunol.

[32] Liu W, Cai MJ, Zheng CC, Wang JX, Zhao XF (2014). Phospholipase Cγ1 connects the cell membrane pathway to the nuclear receptor pathway in insect steroid hormone signaling. J Biol Chem.

[33] Zhou X, Riddiford LM (2002). Broad specifies pupal development and mediates the 'status quo' action of juvenile hormone on the pupal-adult transformation in *Drosophila* and *Manduca*. Development.

[34] Chang SC, Heacock PN, Clancey CJ, Dowhan W (1998). The PEL1 gene (renamed PGS1) encodes the phosphatidylglycero-phosphate synthase of *Saccharomyces cerevisiae*. J Biol Chem.

[35] Fernandez S, Homann MJ, Henry SA, Carman GM (1986). Metabolism of the phospholipid precursor inositol and its relationship to growth and viability in the natural auxotroph *Schizosaccharomyces pombe*. J Bacteriol.

[36] Carman GM, Han GS (2009). Regulation of phospholipid synthesis in yeast. J Lipid Res.

[37] Pereira MG, Visbal G, Costa TFR, Frases S, de Souza W, Atella G (2018). *Trypanosoma cruzi epimastigotes* store cholesteryl esters in lipid droplets after cholesterol endocytosis. Mol Biochem Parasitol.

[38] Cases S, Novak S, Zheng YW, Myers HM, Lear SR, Sande E (1998). ACAT-2, a second mammalian acyl-CoA:cholesterol acyltransferase. Its cloning, expression, and characterization. J Biol Chem.

[39] Lehmann M (2021). Diverse roles of phosphatidate phosphatases in insect development and metabolism. Insect Biochem Mol Biol.

[40] Qi J, Lang W, Giardino E, Caldwell GW, Smith C, Minor LK (2010). High-content assays for evaluating cellular and hepatic diacylglycerol acyltransferase activity. J Lipid Res.

[41] Dunn PE, Bohnert TJ, Russell V (1994). Regulation of antibacterial protein synthesis following infection and during metamorphosis of *Manduca sexta*. Ann N Y Acad Sci.

[42] Wheeler R, Chevalier G, Eberl G, Gomperts BI (2014). The biology of bacterial peptidoglycans and their impact on host immunity and physiology. Cell Microbiol.

[43] Moll RM, Romoser WS, Modrzakowski MC, Moncayo AC, Lerdthusnee K (2001). Meconial peritrophic membranes and the fate of midgut bacteria during mosquito (Diptera: Culicidae) metamorphosis. J Med Entomol.

[44] Hegedus DD, Toprak U, Erlandson M (2019). Peritrophic matrix formation. J Insect Physiol.

[45] Xu Q, Lu A, Xiao G, Yang B, Zhang J, Li X (2012). Transcriptional profiling of midgut immunity response and degeneration in the wandering silkworm, *Bombyx mori*. PLoS ONE.

[46] Russell VW, Dunn PE (1991). Lysozyme in the midgut of *Manduca sexta* during metamorphosis. Arch Insect Biochem Physiol.

[47] Nash JA, Ballard TN, Weaver TE, Akinbi HT (2006). The peptidoglycan-degrading property of lysozyme is not required for bactericidal activity in vivo. J Immunol.

[48] Liu PP, Wei Z, Cheng ZH, Wang XW (2022). Small immune effectors coordinate peptidoglycan-derived immunity to regulate intestinal bacteria in shrimp. PLoS Pathog.

[49] Liu FF, Liu Z, Li H, Zhang WT, Wang Q, Zhang BX (2022). CTL10 has multiple functions in the innate immune responses of the silkworm, *Bombyx mori*. Dev Comp Immunol.

[50] Zhang Y, Ai H, Wang Y, Zhang P, Du L, Wang J (2022). A pattern recognition receptor C-type lectin TcCTL14 contributes to immune response and development in the red flour beetle, *Tribolium castaneum*. Insect Sci.

[51] Charroux B, Capo F, Kurz CL, Peslier S, Chaduli D, Viallat-Lieutaud A (2018). Cytosolic and secreted peptidoglycan-degrading enzymes in *Drosophila* respectively control local and systemic immune responses to microbiota. Cell Host Microbe.

[52] Merkey AB, Wong CK, Hoshizaki DK, Gibbs AG (2011). Energetics of metamorphosis in *Drosophila melanogaster*. J Insect Physiol.

[53] Zhao X, Karpac J (2020). The *Drosophila* midgut and the systemic coordination of lipid-dependent energy homeostasis. Curr Opin Insect Sci.

[54] Martínez BA, Hoyle RG, Yeudall S, Granade ME, Harris TE, Castle JD (2020). Innate immune signaling in *Drosophila* shifts anabolic lipid metabolism from triglyceride storage to phospholipid synthesis to support immune function. PLoS Genet.

[55] Li S, Yu X, Feng Q (2019). Fat body biology in the last decade. Annu Rev Entomol.

[56] Wang X, Hou Y, Saha TT, Pei G, Raikhel AS, Zou Z (2017). Hormone and receptor interplay in the regulation of mosquito lipid metabolism. Proc Natl Acad Sci U S A.

[57] Kibirige D, Ssekitoleko R (2013). Endocrine and metabolic abnormalities among HIV-infected patients: a current review. Int J STD AIDS.

[58] Mason CJ, Ray S, Shikano I, Peiffer M, Jones AG, Luthe DS (2019). Plant defenses interact with insect enteric bacteria by initiating a leaky gut syndrome. Proc Natl Acad Sci U S A.

[59] Zhang MM, Luo LL, Liu Y, Wang GJ, Zheng HH, Liu XS (2022). Calcium and integrin-binding protein 1-like interacting with an integrin α-cytoplasmic domain facilitates cellular immunity in *Helicoverpa armigera*. Dev Comp Immunol.

[60] Yang M, Li G, Yu L, Du S, Jiang D, Chu X (2023). Temperature and metal ions regulate larval diapause termination via the 20-hydroxyecdysone and juvenile hormone pathways in *Monochamus alternatus*. Pest Manag Sci.

[61] Wang JL, Liu XS, Zhang Q, Zhao HB, Wang YF (2012). Expression profiles of six novel C-type lectins in response to bacterial and 20E injection in the cotton bollworm (*Helicoverpa armigera*). Dev Comp Immunol.

[62] Peng H, Chen B, Wei W, Guo S, Han H, Yang C (2022). N6-methyladenosine (m6A) in 18S rRNA promotes fatty acid metabolism and oncogenic transformation. Nat Metab.

[63] Sequencing of bacterial 16S rRNA gene from the midgut of *Helicoverpa armigera*. NCBI BioProject accession: PRJNA1051296. https://www.ncbi.nlm.nih.gov/bioproject/PRJNA1051296 (2023).

[64] Transcriptome sequencing of the midgut from *Helicoverpa armigera* larvae. NCBI BioProject accession: PRJNA1042681. https://www.ncbi.nlm.nih.gov/bioproject/PRJNA1042681 (2023).

[65] Transcriptome sequencing of *Helicoverpa armigera* midgut treated with dsCD209 or dsGFP. NCBI BioProject accession: PRJNA1043398. https://www.ncbi.nlm.nih.gov/bioproject/PRJNA1043398 (2023).

[66] Wang J. A CTL-Lys immune function maintains insect metamorphosis by preventing gut bacterial dysbiosis and limiting opportunistic infections. Mendeley Data. 10.17632/jd4w4wp4hh.1 (2023). 10.1186/s12915-024-01855-8 PMC10918859 38448930

