# Supplementary material for: A CTL − Lys immune function maintains insect metamorphosis by preventing gut bacterial dysbiosis and limiting opportunistic infections
Source: BMC Biol. 2024 Mar 6;22:54. doi: 10.1186/s12915-024-01855-8 (PMC10918859; doi:10.1186/s12915-024-01855-8)
Supplement: Supplementary file 2 — Additional file 2: Figure S1. Quantification of total bacteria in the midgut (A) and hemolymph (B) in the feeding and wandering stages. Figure S2. 20E induces the expression of CD209 and Lys1 in the midgut. Figure S3. Binding of CD209 to L. plantarum independent of Ca2+. Figure S4. Conserved protein sequence of H. armigera Lys1 with C-type Lys. Figure S5. Efficiency of gut bacterial elimination. Figure S6. Suppression in 20E signaling in CD209- or Lys1-depleted larvae, as well as in L. plantarum-injected or L. plantarum-fed larvae. Figure S7. 20E treatment shortens the duration of sixth-instar in CD209- or Lys1-depleted larvae, as well as in L. plantarum-injected or L. plantarum-fed larvae. Figure S8. Metabolomics analysis of hemolymph samples from H. armigera larvae injected with L. plantarum (LP) or PBS. [file 12915_2024_1855_MOESM2_ESM.docx]

**Additional file 2 for “****A CTL−Lys immune function maintains insect metamorphosis by preventing gut bacterial dysbiosis and limiting opportunistic infections”**

Pei Xiong *et al*.

Corresponding author: Jia-Lin Wang, [jlwang@ccnu.edu.cn](mailto:jlwang@ccnu.edu.cn)


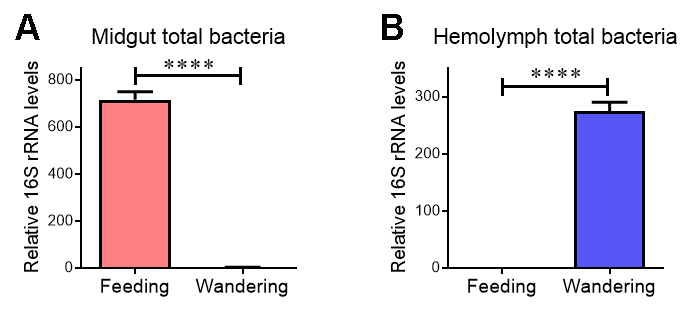


**Figure S1.** Quantification of total bacteria in the midgut (**A**) and hemolymph (**B**) in the feeding and wandering stages. Quantification was by 16S rRNA gene-based qPCR analysis. Statistical differences were analyzed using Student’s *t* test (*****p* < 0.0001).


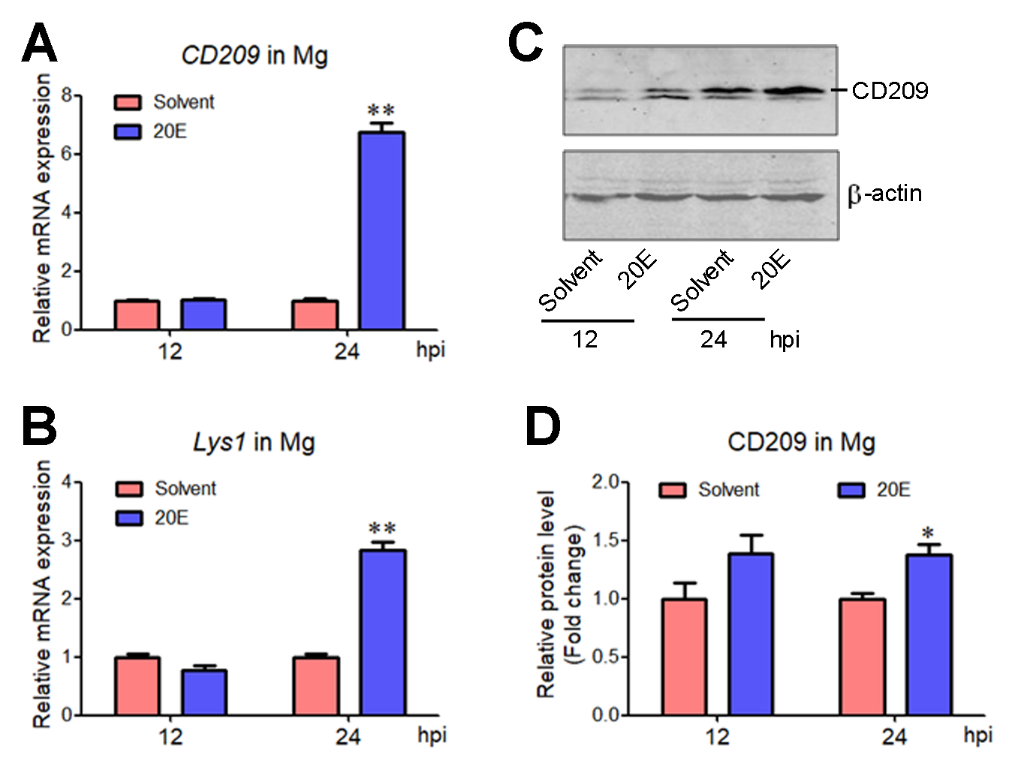


**Figure S2.** 20E induces the expression of CD209 and Lys1 in the midgut. **A**, **B** Analysis of *CD209* (**A**) and *Lys1* (**B**) transcripts in the midgut (Mg) at 12 and 24 h post-20E injection (hpi) using RT-qPCR. **C** Western blot analysis of CD209 proteins in the Mg at 12 and 24 h post-20E injection. Antiserum against CD209 or β-actin was used for detection. **D** Quantification of the relative level of CD209 in the Mg detected using western blotting. Statistical differences were analyzed using Student’s *t* test (**p* < 0.05 and ***p* < 0.01).


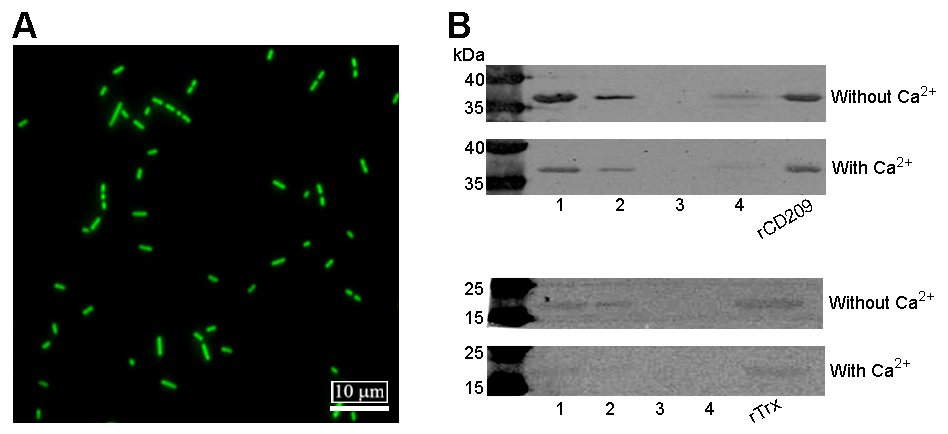


**Figure S3.** Binding of CD209 to *L. plantarum* independent of Ca^2+^. **A** Morphological characterization of *L. plantarum*. The bacteria (FITC-stained) were photographed under a fluorescence microscope. Scale bar = 10 μm. **B** Binding of rCD209 to *L. plantarum* in the presence or absence of Ca^2+^. Bacteria were incubated with rCD209 or rTrx (negative control), followed by three PBS washed (lanes 1–3) and SDS elution (lane 4). The rCD209 or rTrx was loaded as control. Anti-His antibody was used for detection.


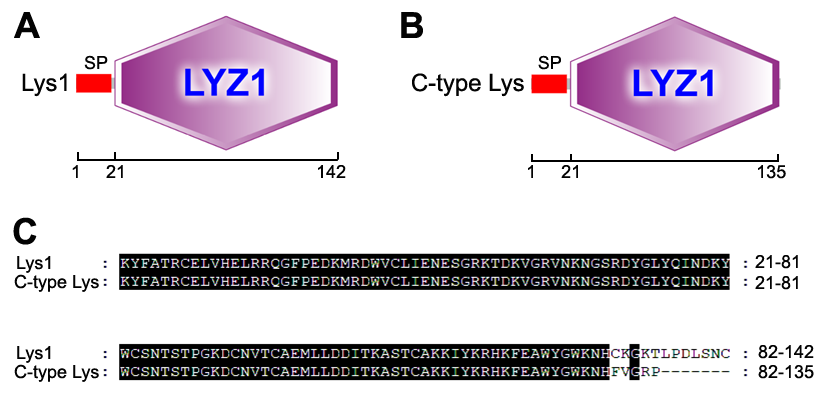


**Figure S4.** Conserved protein sequence of *H. armigera* Lys1 with C-type Lys. **A**, **B** Predicted domain architecture of Lys1 (**A**) and C-type Lys (**B**). Characterization of a signal peptide (SP) and a lysozyme type C (LYZ1) domain from http://smart.embl.de/ website. **C** Sequence alignment of *H. armigera* Lys1 and C-type Lys mature peptide. Identical amino acids are shaded in black.


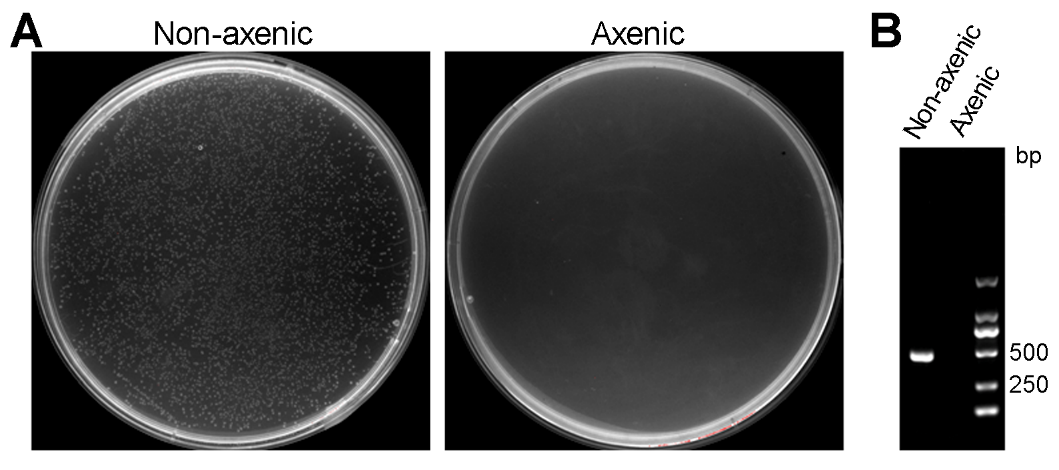


**Figure S5.** Efficiency of gut bacterial elimination. **A** Culture of gut homogenates on LB agar plates. **B** PCR amplification of a fragment of bacterial 16S rRNA gene.


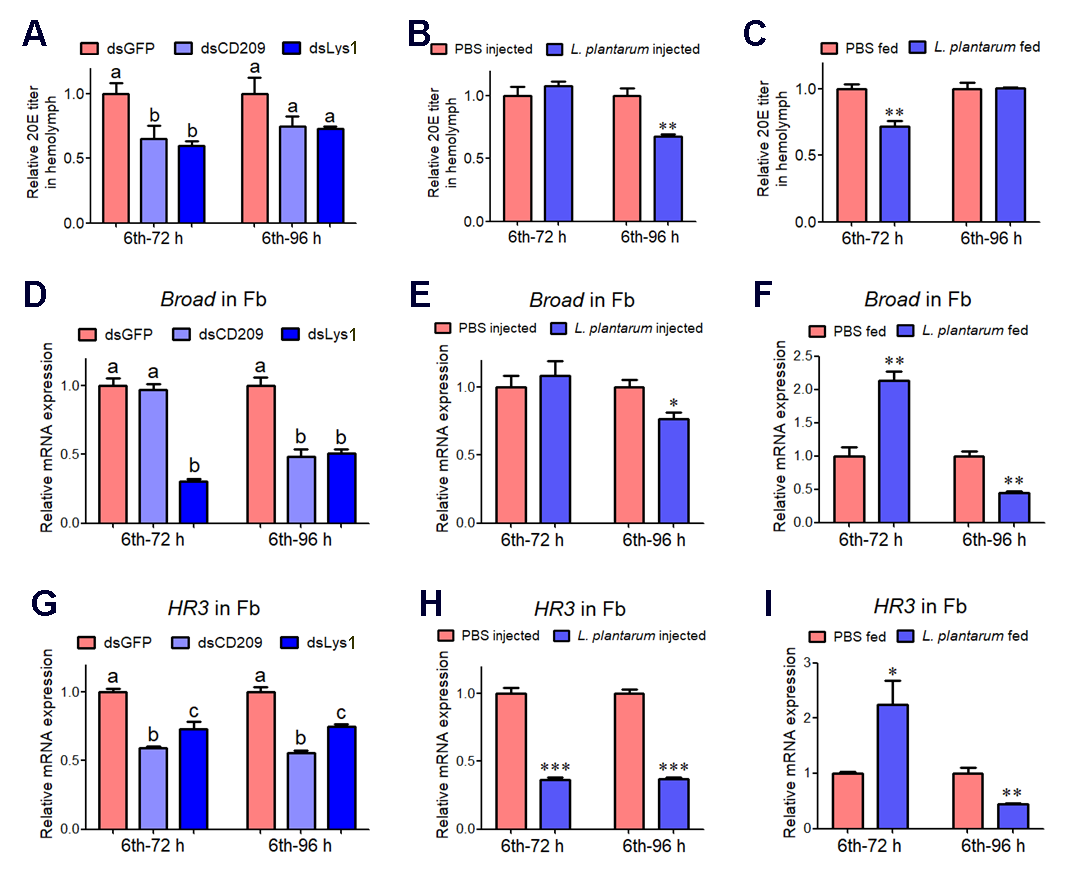


**Figure S6.** Suppression in 20E signaling in CD209- or Lys1-depleted larvae, as well as in *L. plantarum*-injected or *L. plantarum*-fed larvae. **A** Measurement of relative 20E titers in the hemolymph of ds*CD209*-, ds*Lys1*-, or ds*GFP*-injected larvae. **B**, **C** Measurement of relative 20E titers in the hemolymph of *L. plantarum*-injected larvae (**B**) and *L. plantarum*-fed larvae (**C**). PBS-injected or fed as control. **D**, **G** Expression profiles of *broad* (**D**) and *HR3* (**G**) in the fat body (Fb) of ds*CD209*-, ds*Lys1*-, or ds*GFP*-injected larvae. **E**, **H** Expression profiles of *broad* (**E**) and *HR3* (**H**) in the Fb of *L. plantarum*- or PBS-injected larvae. **F**, **I** Expression profiles of *broad* (**F**) and *HR3* (**I**) in the Fb of *L. plantarum*- or PBS-fed larvae. Hemolymph and Fb were collected from sixth-instar larvae at 72 h PE (6th-72 h) and 96 h PE (6th-96 h). Statistical differences were analyzed using Student’s *t* test (**p* < 0.05, ***p* < 0.01, and ****p* < 0.001) or one-way ANOVA followed by Tukey’s multiple comparison test. Different characters above the bars indicate significant differences.


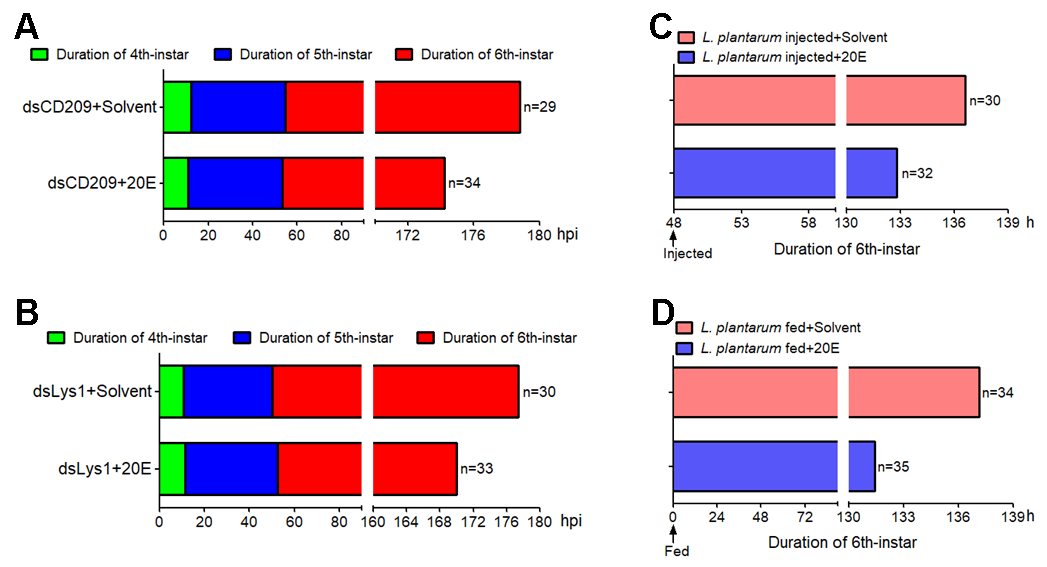


**Figure S7.** 20E treatment shortens the duration of sixth-instar in CD209- or Lys1-depleted larvae, as well as in *L. plantarum*-injected or *L. plantarum*-fed larvae. Larvae pre-injected with ds*CD209* (**A**), ds*Lys1* (**B**), or *L. plantarum* (**C**), as well as fed by *L. plantarum* (**D**), were injected with 20E or DMSO (solvent control) at 72 h PE of the sixth-instar. The duration of each instar was measured individually and the mean value was calculated.


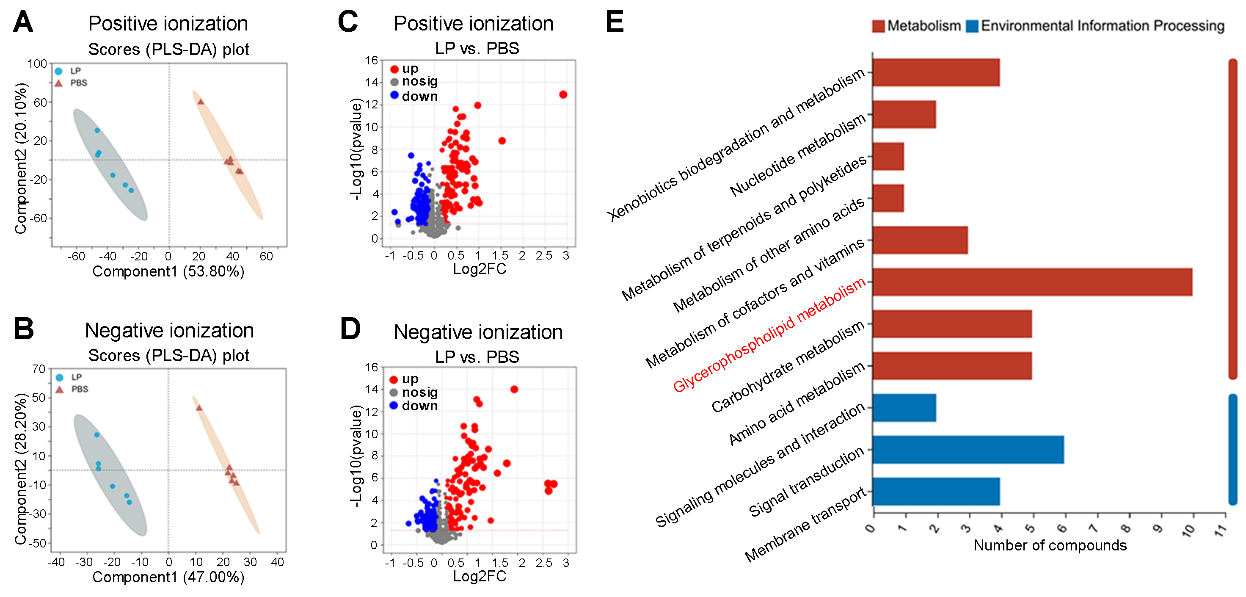


**Figure S8.** Metabolomics analysis of hemolymph samples from *H. armigera* larvae injected with *L. plantarum* (LP) or PBS. **A**, **B** PLS-DA of positive (**A**) and negative (**B**) ionization datasets for larvae injected with *L. plantarum* or PBS. **C**, **D** Volcano map of all differential metabolites in positive (**C**) and negative (**D**) ionization models (*p* < 0.05). The *p* values were determined using Student’s *t* test. FC, fold change; nosig, no significant change. **E** KEGG pathway classification of differential metabolites between the hemolymph samples from *L. plantarum*- and PBS-injected larvae. The name of each KEGG pathway is indicated on the left, and the number of compounds is shown below. The pathway involved in glycerophospholipid metabolism (red) is highlighted.
