# Supplementary figures and images for: A CTL − Lys immune function maintains insect metamorphosis by preventing gut bacterial dysbiosis and limiting opportunistic infections
Source: BMC Biol. 2024 Mar 6;22:54. doi: 10.1186/s12915-024-01855-8 (PMC10918859; doi:10.1186/s12915-024-01855-8)

**Figure 2D**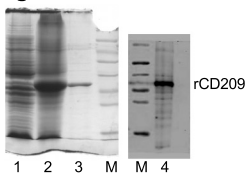**Figure 2F**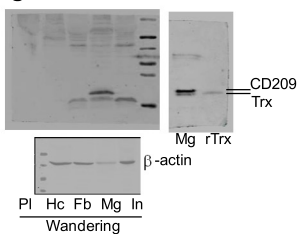**Figure 3D**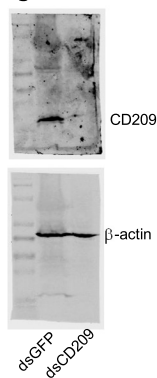**Figure 4D**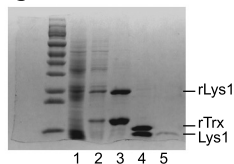**Figure S2C**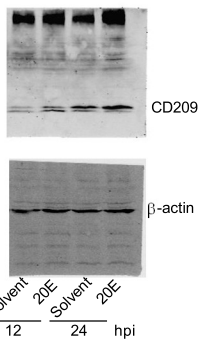**Figure S3B**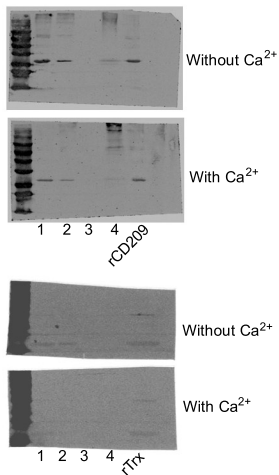**Figure S5B**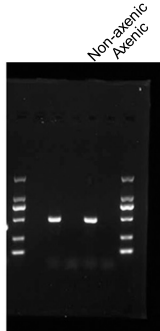

Supplement: Supplementary file 3 — Additional file 3. Original images of western blot and PCR gel. [file 12915_2024_1855_MOESM3_ESM.pdf]
